# Robust and reusable self-organized locomotion of legged robots under adaptive physical and neural communications

**DOI:** 10.3389/fncir.2023.1111285

**Published:** 2023-03-31

**Authors:** Tao Sun, Zhendong Dai, Poramate Manoonpong

**Affiliations:** ^1^Neurorobotics Technology for Advanced Robot Motor Control Lab, The College of Mechanical and Electrical Engineering, Nanjing University of Aeronautics and Astronautics, Nanjing, China; ^2^Wearable Systems Lab, School of Mechanical Engineering, Shanghai Jiao Tong University, Shanghai, China; ^3^Bio-Inspired Robotics and Neural Engineering Lab, School of Information Science and Technology, Vidyasirimedhi Institute of Science and Technology, Rayong, Thailand

**Keywords:** self-organized locomotion, neural control, physical communication, neural communication, walking robots

## Abstract

**Introduction:**

Animals such as cattle can achieve versatile and elegant behaviors through automatic sensorimotor coordination. Their self-organized movements convey an impression of adaptability, robustness, and motor memory. However, the adaptive mechanisms underlying such natural abilities of these animals have not been completely realized in artificial legged systems.

**Methods:**

Hence, we propose adaptive neural control that can mimic these abilities through adaptive physical and neural communications. The control algorithm consists of distributed local central pattern generator (CPG)-based neural circuits for generating basic leg movements, an adaptive sensory feedback mechanism for generating self-organized phase relationships among the local CPG circuits, and an adaptive neural coupling mechanism for transferring and storing the formed phase relationships (a gait pattern) into the neural structure. The adaptive neural control was evaluated in experiments using a quadruped robot.

**Results:**

The adaptive neural control enabled the robot to 1) rapidly and automatically form its gait (i.e., self-organized locomotion) within a few seconds, 2) memorize the gait for later recovery, and 3) robustly walk, even when a sensory feedback malfunction occurs. It also enabled maneuverability, with the robot being able to change its walking speed and direction. Moreover, implementing adaptive physical and neural communications provided an opportunity for understanding the mechanism of motor memory formation.

**Discussion:**

Overall, this study demonstrates that the integration of the two forms of communications through adaptive neural control is a powerful way to achieve robust and reusable self-organized locomotion in legged robots.

## 1. Introduction

Some animals (e.g., wild cattle) can perform adaptive locomotion within minutes of being born.^1^ Such animals can move robustly in the natural environment and memorize their self-organized locomotion. The locomotion is rapidly formed through dynamic body-environment interactions that alter their neural locomotion control circuits comprising genetically encoded structures (Dickinson, 2000; Kullander et al., 2003). Furthermore, they can robustly walk even when experiencing perturbations or missing sensory feedback (Grillner and Zangger, 1984; MacKay-Lyons, 2002). However, such a rapidly and automatically generated (self-organized) robust locomotion with motor memory has not been fully realized in legged robots. Although some robots have exhibited certain excellent locomotion behaviors, their designs are typically based on an engineering approach, which often requires accurate kinematic models (Raibert et al., 2008; Hutter et al., 2016; Semini et al., 2017; Bledt et al., 2018). Moreover, the approach is difficult to relate to its biological counterpart to better understand and realize an adaptive interlimb coordination for self-organized robot locomotion.

In contrast to the engineering approach, biologically inspired approaches based on underlying biological principles, such as central pattern generators (CPGs) (Marder and Bucher, 2001) and reflex chains (Lundberg, 1979) with sensory feedback, have been implemented on various robots (Kimura et al., 2007; Ijspeert, 2008; Ajallooeian et al., 2013; Tran et al., 2014; Yu et al., 2014; Aoi et al., 2017; Lodi et al., 2020). Some of these robots have performed adaptive motor patterns without kinematic models. However, their versatile behavior often requires elaborate preprogrammed rules for providing specific connections among (neural) control networks or units (Steingrube et al., 2010; Fukuoka and Kimura, 2014; Fukui et al., 2019). For example, Fukuoka et al. developed a neural system with predefined coupled CPGs and reflex mechanisms for a series of Tekken robots (Kimura et al., 2007; Fukuoka and Kimura, 2014). The robots controlled by the neural system can dynamically generate locomotion on natural terrain. Based on reflex mechanisms and biological observation of stick insects, Cruse et al. proposed Walknet, a set of specific behavioral rules with neural networks for legged locomotion (Cruse et al., 1998). The specific rules are considered as predefined neural-wired connections (neural communication) between networks. Generally, these approaches entail designing interlimb coordination through biological observation before transferring or implementing the locomotion control to robots. This resulted in limitations pertaining to interlimb coordination in terms of real-time adaptation and flexibility. An alternative solution for autonomously creating locomotion control is the use of machine learning (ML), which has continued to become more sophisticated and practical over the past few decades.

Some ML techniques, such as reinforcement learning (RL) (Nakamura et al., 2007; Cully et al., 2015; Heess et al., 2017; Hwangbo et al., 2019; Ishige et al., 2019; Jones et al., 2020; Thor et al., 2020) and evolutionary algorithms (EA) (Juang and Yeh, 2018), have been proposed to automatically tune a neural control network for robust robot locomotion. Although these approaches may enable robot agility, complex motor skills, and adaptability to various environments, they typically have a time-cost learning process and a sim-to-real transfer gap. This is because the RL/EA-based robot neural control network, unlike the genetically encoded neural network of animals, is typically trained from a random structure (or scratch). Therefore, animals spend their first moments of life fine-tuning the network, rather than learning it from scratch (Kullander et al., 2003), which renders it difficult to relate the ML-based control methods to animal locomotion control mechanisms or principles.

To address this problem, Owaki et al. introduced a simple but effective Tegotae-based control approach (Owaki et al., 2012). They demonstrated that distributed decoupled CPGs with local ground reaction force (GRF) feedback could rapidly facilitate self-organized locomotion, similar to that of animals, through dynamic body-environment interactions (physical communication) (Dallmann et al., 2017). In other words, the GRF feedback provides a communication channel for the CPGs through a physical body, enabling the channel to indirectly reflect the motion state of other legs. Compared to other traditional learning strategies, the control scheme using the physical communication requires only fewer steps to obtain a stable self-organized gait. Moreover, the control eliminates the gap between the simulation and physical world because it does not require numerous iterations and thus can be directly implemented on real robots. However, the effectiveness of the physical communication for locomotion generation significantly depends on the uninterrupted functionality of the load-sensing feedback (i.e., the GRF). Furthermore, the feedback gain must be predefined and stable locomotion convergence cannot be guaranteed if the sensory feedback encounters disturbances or produces an unstable pattern (Sun et al., 2021b). Another drawback is the impossibility of storing the generated locomotion (i.e., no motor memory). In other words, the Tegotae-based approach does not possess the capability of mammals to store generated locomotion patterns in their spinal cords (Wolpaw, 2010). Such a memory can be obtained through formed connections between CPGs or neural control units. The connections will essentially create neural communication paths and couplings that are beneficial for factors such as locomotion recovery.

To overcome the shortcomings of the Tegotae-based approach, we proposed adaptive neural control with adaptive physical and neural communications (APNC). The proposed control has the following distinct features: 1) it employs an online learner (dual-rate learning Smith et al., 2006) to automatically tune sensory feedback gains, thereby creating more adaptive physical communication (APC), and 2) it combines the APC with a type of novel adaptive neural communication (ANC) algorithm for robust and reusable self-organized locomotion on even (as shown using Tegotae) and uneven terrains. The ANC uses a fast online learning strategy that can acquire and estimate the phase relationships among leg movements originally generated through physical communication. Subsequently, it automatically creates neural couplings between the distributed decoupled CPGs such that they can be stably synchronized.

The neural couplings adaptively stabilize the locomotion pattern and act as motor memory for reuse. Compared to the typical, predefined neural connections and reflex approaches (Collins and Richmond, 1994; Kimura et al., 2007; Ajallooeian et al., 2013; Fukuoka and Kimura, 2014; Tran et al., 2014; Yu et al., 2014; Aoi et al., 2017), the APNC (a combination of APC and ANC) provides greater flexibility and adaptability for locomotion generation because the locomotion simply emerges from the interactions between the robot and its environment (Hülse et al., 2007). Moreover, the locomotion generation is fast and robust to disturbances because of the adaptive neural couplings formed by the ANC. Furthermore, the ANC also enables a robot to memorize the self-organized motor pattern within a few seconds.

The key specific problems the study addressed are 1) how to achieve robust self-organized locomotion under various situations (including sensor malfunction, uneven terrain, noisy feedback, leg damage, carrying a payload, different locomotion speeds, and different control update frequencies) and 2) how to store or transfer formed locomotion into a neural structure as motor memory for reusable locomotion or locomotion recovery. These two main issues have not been fully solved or addressed by current state-of-the-art fast self-organized locomotion control methods (i.e., Tegotae-based control Kano et al., 2017; Owaki et al., 2017, 2021 and phase resetting (PR)-based control Nomura et al., 2009; Aoi et al., 2011, 2012, 2021; Ambe et al., 2021). Furthermore, this study is also significantly different from our previous study which focused only on adaptive joint (intralimb) coordination with a fixed or predefined gait (i.e., predefined interlimb coordination) for slope walking (Sun et al., 2021a). Accordingly, the key contributions of this work include the following: 1) providing a novel integrative approach of physical and neural communications *via* adaptive neural control for fast, robust, and reusable self-organized locomotion (or self-organized, adaptive interlimb coordination); 2) demonstrating the effectiveness and robustness of the adaptive neural control and its motor memory through a quadruped robot in both simulated and real-world environments under the various conditions, as well as comparing the proposed neural control method to the state-of-the-art methods; 3) gaining a better understanding of the interaction between sensory feedback, CPGs, and neural mechanisms for rapidly generating adaptive, robust, and reusable locomotion; 4) introducing a control architecture that can serve as a basis for developing “GEneral NEural control for Self-organIzed emergent behavior of legged/limbed Systems (GENESIS).”

## 2. Adaptive neural control

**Figure 1:**
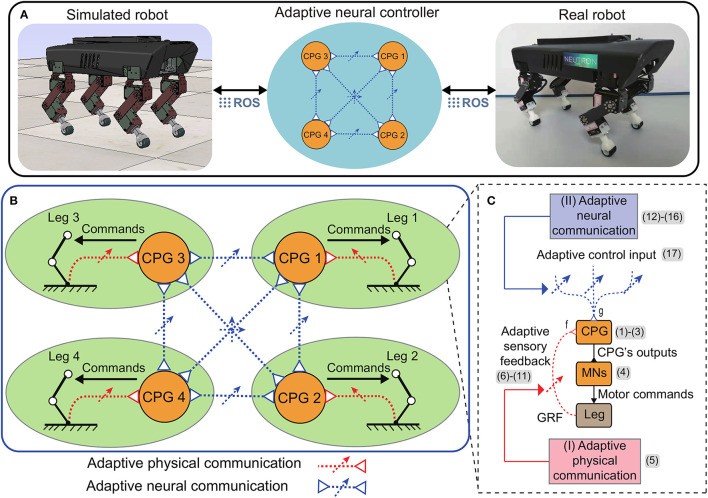
**(A)** Overview of the adaptive neural control implemented on a quadruped robot in both simulation and real world (see the Supplementary material for more details of the simulated and real robot setups). **(B)** The control is based on decoupled CPG-based control circuits and the adaptive physical and neural communications (APC and ANC). The number (1–17) represent the equation numbers. **(C)** The main ingredients of each local CPG-based control circuit includes a SO(2) CPG (Pasemann et al., 2003) to produce rhythmic signals, motor neurons (MNs), and two adaptive modulation mechanisms (adaptive sensory feedback and adaptive control input).

Here, we propose the adaptive neural control (called APNC-based control) that enables robots to achieve robust and reusable self-organized locomotion. As shown in Figure 1A, in this study, the control was applied to Lilibot, a quadruped robot (Sun et al., 2020) (see also Supplementary Figure S1), in both simulated and real-world environments through the robot operation system (ROS, see Supplementary Figure S2). The control consists of four identical local CPG-based neural circuits that send motor commands to the legs (Figure 1B). The CPGs are adaptively coupled *via* the APNC. The APC is derived from the interaction between the body dynamics and environment through the GRF feedback of each leg. During the interaction, the sensory feedback gain is adjusted online (Figure 1B, red dashed lines) to quickly achieve stable interlimb coordination. Subsequently, the interlimb coordination (the phase relationships among the CPGs) is maintained by the ANC through neural couplings (Figure 1B, blue dashed lines). The neural couplings of the ANC are adaptively controlled online based on the performance of the APC. After the parameters of the APC and ANC converge, the control effectively generates and stabilizes the self-organized coordinated motor commands for all leg movements.

Specifically, each CPG-based neural circuit (Figure 1C) possesses two main components. The first component is a CPG to produce rhythmic signals that are subsequently transferred to drive joint movements through the second component consisting of motor neurons (MNs). To coordinate the signals, the CPG is modulated by integrating the adaptive sensory feedback and adaptive control input. The adaptive sensory feedback and adaptive control input are achieved using the APC and ANC, respectively. The two adaptive communication mechanisms are described in detail below.

### 2.1. Adaptive physical communication

A fundamental problem posed by legged locomotion with multiple degrees of freedom is interlimb coordination, whereby the phase relationships between the leg movements must be defined such as to form a stable gait. Instead of predefining the relationships, as is typically done in most locomotion control methods (Collins and Richmond, 1994; Ijspeert et al., 2007; Kimura et al., 2007; Ijspeert, 2008; Zeng et al., 2018), we employed a self-organized interlimb coordination strategy through physical communication, as proposed by Owaki et al. (2012) and Owaki and Ishiguro (2017). This approach is flexible and transferable to different types of legged robots. The strategy employs distributed, decoupled CPG-based control and utilizes GRF feedback to automatically adjust CPG phase relationships. However, in Owaki et al. (2012) and Owaki and Ishiguro (2017), the GRF feedback gains to their CPGs were manually adjusted or empirically selected.

**Figure 2:**
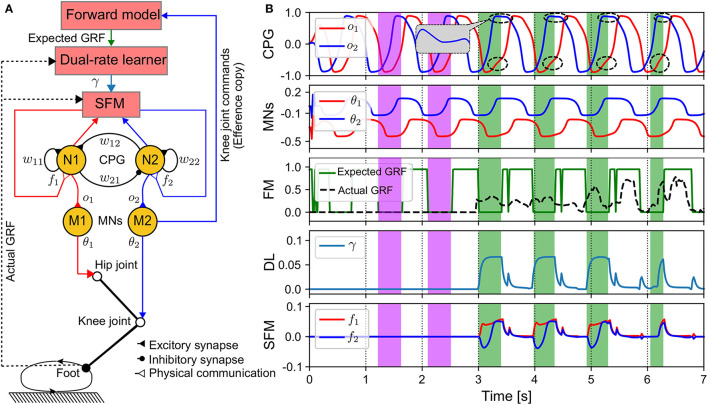
APC mechanism of one leg. **(A)** Diagram of components realizing the APC. The DL can produce the adaptive gain depending on the error between the actual and expected GRF signals. The expected GRF signal is obtained from the forward model, which translates the motor command of the knee joint (efference copy) into the expected foot state (swing or stance state). **(B)** Outputs of the components. The purple and green color areas show initial (no adaptation) and strongly adaptation periods, respectively. The abrupt changes of the expected GRF at around 3.5 s, 4.5 s, 5.5 s to 0.0 are due to a brief ascending of the CPG output during the expected descending periods. The control parameters used in this test are listed in Supplementary Table S2. In this investigation, the SFM is activated to adaptively modulate the CPGs after 3 s and the stable gait is formed within 3–4 s.

Here, we propose an adaptive physical communication (APC) mechanism by determining the physical communication strategy with sensory adaptation (Wark et al., 2007) that is an adaptive mechanism based on error-based learning for automatic sensory feedback gain adjustment. The adaptive mechanism allows the APC to modify (or strengthen) the sensory feedback gain to strongly transmit the actual GRF signal to adjust the CPG's activations when the leg receives the GRF signal in the swing phase (i.e., the actual GRF is larger than the expected GRF, see green areas in Figure 2), while during the stance phase the feedback strength is reduced since the actual GRF is basically smaller than the expected GRF (Figure 2). As a result, during this phase the CPG's activations will be slightly adjusted through the decay adaptive gain (see the first right terms in Equations (7), (8). As demonstrated in this study, this adaptation strategy intriguingly results in fast and stable self-organized locomotion.

The architecture of the APC and its modules' outputs can be seen in Figure 2. The APC requires five components including: a CPG, motor neurons (MNs), a sensory feedback mechanism (SFM), a dual-rate learner (DL), and a forward model (FM) (Figure 2A). The outputs of the CPG are transferred to the MNs (M1 and M2), which are linear neurons with scaling factors that shape the CPG output signals (see Figure 2B). The outputs of the MNs are transmitted to the hip and knee motors as the inputs of the position control for driving the motors. The M1 output is fetched to the FM that can estimate an expected GRF. The expected and actual GRFs are transferred to the DL.

The DL outputs (*K*_*f,s*_(*n*)) are used to determine the strength of the sensory feedback. From Figure 2B, during the first period (3 s), γ is zero, and hence the SFM outputs (*f*_1,2_(*n*)) are also zero. This means that there are no sensory signals to affect the CPGs' activations. This occurs because the actual GRF is zero at the initial period (see the purple areas and the FM plot in Figure 2B). After 3 s, the SFM is activated to strongly modulate the activations of the CPGs *via* *f*_1,2_(*n*) (see the dashed circles in Figure 2B) when the actual GRF is larger than the expected GRF (see green areas in Figure 2B).

#### 2.1.1. Basic rhythmic pattern generation

For the rhythmic pattern generation, the CPG is realized by a neural SO(2) oscillator^2^ (Pasemann et al., 2003). The SO(2) oscillator has two fully connected standard non-spiking neurons (N1 and N2, see Figure 2A), both of which have a sigmoid transfer function. The activation *a*_*i*_ and output *o*_*i*_ of each neuron are provided by Equations (1), (2):


(1) $$\begin{array}{l} a_{i}\left(n+1\right)=\overset{2}{\underset{j=1}{\sum }}w_{ij}o_{j}\left(n\right)-f_{i}\left(n\right),i=1,2, \end{array}$$

(2) $$\begin{array}{l} o_{i}\left(n\right)=\tanh\left(a_{i}\left(n\right)\right),i=1,2, \end{array}$$

where *w*_*ij*_ is the synaptic weight of the connection from the *j*th neuron to the *i*th neuron. *n* indicates a time step of discrete-time equations. One time step is related to 1/update frequency. All the weights and *MI* are defined using Equation (3).


(3) $$\begin{array}{l} \begin{array}{c} w_{12}=0.21+\text{MI},\;w_{21}=-w_{12},\;w_{11,22}=1.4, \end{array} \end{array}$$

where *MI* is the modulatory input of the CPG synaptic weights (Manoonpong et al., 2013). Using different *MI* values lead to different CPG frequencies and, as a result, different walking frequencies. Note that the default synaptic weights of 0.21 and 1.4 are selected from the parameter domains that stay beyond a Neimark-Sacker bifurcation where periodic or quasi-periodic attractors exist (as investigated in Pasemann et al., 2003). This allows the CPG to produce basic periodic signals at a very low frequency, even when the *MI* value is zero. In Equation (1), *f*_*i*_(*n*) represents the adaptive sensory feedback term that is induced by the APC to adaptively modulate the CPG's phase.

The outputs of the CPG are transferred to the MNs (M1 and M2), which are linear neurons with scaling factors that shape the CPG output signals. The outputs of the MNs are transmitted to the hip and knee motors as the inputs of the position control for driving the motors. The MNs can be represented as follows:


(4) $$\begin{array}{l} \theta_{i}\left(n\right)=a_{i}o_{i}\left(n\right)+b_{i},i=1,2, \end{array}$$

Where *o*_*i*_(*n*) is the output of the *i*th neuron in a CPG and *a*_*i*_ and *b*_*i*_ are the slope and offset of the linear transformation of the MNs, respectively. θ_*i*_(*n*) is the output of the corresponding MN. The hip and knee joints extend (clockwise rotation) as θ_*i*_(*n*) increases, while the joints flex (counterclockwise rotation) as θ_*i*_(*n*) decreases.

#### 2.1.2. Adaptive CPG phase modulation

The CPG phase is directly modulated by the SFM that can be described by the following equations:


(5) $$\begin{array}{l} f_{i}\left(n\right)=\{\begin{array}{c} \gamma \left(n\right)F\left(n\right)\cos\left(o_{i}\left(n\right)\right),\;i=1\\ \gamma \left(n\right)F\left(n\right)\sin\left(o_{i}\left(n\right)\right),\;i=2 \end{array}, \end{array}$$

Where *o*_*i*_(*n*) is the output of the *i*th neuron in a CPG. γ(*n*) is an adaptive feedback gain automatically tuned by the DL. *F*(*n*) represents the continuous actual GRF as sensory feedback to the CPG. Note that the GRFs of four legs are normalized to [0, 1) by dividing the measured GRFs with around half of the robot body weight, where zero and nonzero denote a foot in the swing phase and stance phase, respectively. Zero and nonzero denote a foot in the swing phase and stance phase, respectively. The DL is an error-based learning mechanism (Smith et al., 2006) that implements the adaptation of the physical communication.

Specifically, the DL tunes the gain γ(*n*) (see Equation 5) based on the error between the actual and expected GRF signals. Its function is represented by the following equations:


(6) $$\begin{array}{l} e(n)=\{\begin{array}{l} \;F^{a}(n)-F^{e}(n),\;F^{a}(n)-F^{e}(n)>0\\ 0,\;F^{a}(n)-F^{e}(n)\leq 0 \end{array}, \end{array}$$

(7) $$\begin{array}{l} K_{f}\left(n+1\right)=A_{f}K_{f}\left(n\right)+B_{f}e\left(n\right), \end{array}$$

(8) $$\begin{array}{l} K_{s}\left(n+1\right)=A_{s}K_{s}\left(n\right)+B_{s}e\left(n\right), \end{array}$$

(9) $$\begin{array}{l} \gamma \left(n\right)=K_{f}\left(n\right)+K_{s}\left(n\right), \end{array}$$

Where *F*^*a*^(*n*) and *F*^*e*^(*n*) are the actual and expected GRF signals, respectively. *B*_*s*_ and *B*_*f*_ correspond to the slow and fast learning rates of the slow and fast learners, whose slow and fast retention factors are *A*_*s*_ and *A*_*f*_, respectively. The fast learner has a higher learning rate and the slow learner is characterized by a higher retention rate, i.e., *A*_*s*_ > *A*_*f*_ and *B*_*s*_ < *B*_*f*_. The learning parameters used in this study were empirically set to: *A*_*s*_ = 0.99, *A*_*f*_ = 0.57, *B*_*s*_ = 0.0002, and *B*_*f*_ = 0.002.

The expected GRF (*F*^*e*^(*n*)) is mapped from an efference copy of the knee joint by the FM. This is because, in an ideal state, the flexion and extension of the knee joint indicate that the foot should move up into a swing phase and down into a stance phase, respectively. Thus, in the swing phase, the expected GRF is zero; however, in the stance phase, it yields a higher value (> 0). Here, the input to the FM is a sine wave-like pattern (see Figure 2B). The FM can be described as follows:


(10) $$\begin{array}{l} F^{e}\left(n+1\right)=\alpha \left(\rho G\left(n\right)+\left(1-\rho \right)F^{e}\left(n\right)\right), \end{array}$$

(11) $$\begin{array}{l} G\left(n\right)=\{\begin{array}{l} 1,\;\theta_{1}\left(n\right)<=\theta_{1}\left(n-1\right)\\ 0,\;\theta_{1}\left(n\right)>\theta_{1}\left(n-1\right) \end{array}, \end{array}$$

Where *F*^*e*^(*n*) is the expected GRF, α is a scaling factor used to scale the amplitude of the expected GRF such that it matches the actual GRF, ρ is a shaping parameter used to fine-tune the duty factor of the expected GRF. *G*(*n*) is a variable used mainly for switching the expected stance and swing phases depending on θ_1_(*n*), where θ_1_(*n*) is the output of the MN to the hip joint. In the following experiments, the parameters were set to α = 0.9, ρ = 0.99 (see Equation 10). Note that although the selected FM cannot create a complex waveform, a radial basis function network can yield a complex waveform (Thor et al., 2020).

### 2.2. Adaptive neural communication

Although the APC mechanism can automatically generate coordinated motor commands for interlimb or leg coordination, the coordination of the commands is sensitive to any disturbance in the GRFs and cannot be memorized for reuse. Therefore, if there is a strong disturbance or sensory damage, the generated motor commands can become dis-coordinated, leading to unstable locomotion. Furthermore, if the control system is reset, the previously generated coordinated motor commands from the physical communication will no longer be available. Thus, we introduce the ANC to address these problems.

**Figure 3:**
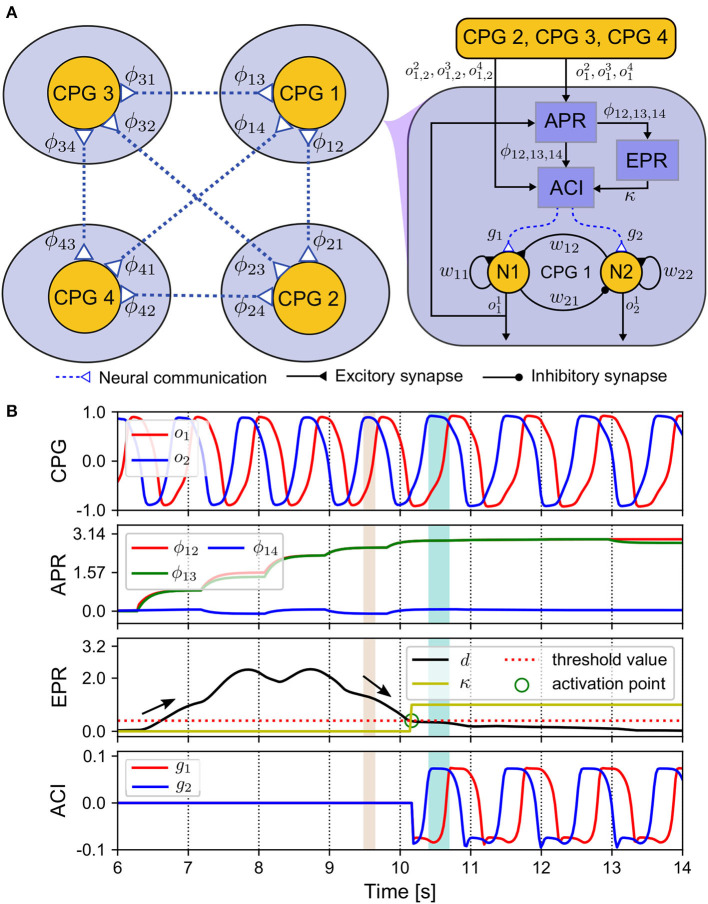
ANC mechanism. **(A)** Diagram of the components realizing the ANC. **(B)** Outputs of these components. The CPG phase relationships (ϕ_12_(*n*), ϕ_13_(*n*), ϕ_14_(*n*)) change from an initial state (0, 0, 0) to a convergence state (π, π, 0) after 4 s. The control parameters used in this test are listed in Supplementary Table S2.

The underlying mechanism of the ANC is to capture the coordinated phases among the CPGs and transfer their stable output patterns into the adaptive neural couplings among the CPGs to memorize the patterns (see Figure 3A). The ANC of one leg includes an acquisition of phase relationships (APR) for observing the CPG phase states, an estimation of the phase relationships (EPR) for triggering the neural communication, and an adaptive control input (ACI) for implementing the coupling effect of the neural communication (Figure 3A).

The outputs of the APR, EPR, and ACI can be seen in Figure 3B. The control system starts at the 6th s. At approximately the 10th s, the variance of relative phase (*d*(*n*)) falls below the threshold, and then the EPR output (κ(*n*)) increases toward 1 from 0. At this point, which we term the activation point, the ANC is triggered. Then, the ACI outputs are introduced to modulate the CPGs. After the 10th s, the relative phases ϕ_12_(*n*) and ϕ_13_(*n*) converge to approximately 3.14 rad, and ϕ_14_(*n*) is approximately zero. Here, the convergence time is approximately 4 s. This indicates that the RH and LF legs move in phase, and in anti-phase with reference to the RF and LH legs. The relative phases of the CPGs of the RH, LF, and LH legs referred to the CPG of the RF leg can be calculated as follows:


(12) $$\begin{array}{l} \phi_{lk}\left(n\right)=\frac{t_{p}^{l}-t_{p}^{k}}{T}\cdot 2\pi , \end{array}$$

Where $t_{p}^{l}$ ($t_{p}^{k}$) is the moment when the neuron N1 output of the *l*th (*k*th) CPG (i.e., *l, k* = 1, 2, 3, 4) attains a peak point within a CPG signal period. *T* is the current period of the CPG signals. Note that when *k* = *l*, ϕ_*lk*_(*n*) = 0 (i.e., the phase shift of a CPG with respect to itself is zero) and ϕ_*lk*_(*n*) = −ϕ_*kl*_(*n*) (i.e., the phase shift of the CPG *k* with respect the CPG *l* is opposite to that of the CPG *l* with respect the CPG *k*).

To transfer the stable pattern (e.g., stable phase relationships ϕ_12_(*n*), ϕ_13_(*n*), and ϕ_14_(*n*)) into the adaptive neural couplings, the first step is to automatically identify and acquire the stable pattern. The CPG relative phases are changeable online before a stable pattern is obtained owing to the APC (see Figure 3B). Thus, in principle, a stable pattern is formed when the relative phases became constant. Therefore, if the distance between the current relative phases and the previous ones is minimal, the pattern is considered stable. Based on this assumption, the EPR is implemented to calculate the stabilization value for the relative phases through the Frobenius norm of the difference between the current and previous average relative phases. Once the Frobenius norm falls below a defined threshold, the pattern of the CPG phase relationships is considered stable, thereby switching on the ANC. This process can be represented as follows:


(13) $$\begin{array}{l} \Phi \left(\text{n}\right)={\left[\begin{array}{cccc} 0 & \phi_{12}\left(n\right) & \phi_{13}\left(n\right) & \phi_{14}\left(n\right) \end{array}\right]}^{T}, \end{array}$$

(14) $$\begin{array}{l} \bar{\Phi \left(\text{n}\right)}=\frac{1}{N}\overset{N}{\underset{i=1}{\sum }}\Phi \left(\text{n }-\text{ i}\right), \end{array}$$

(15) $$\begin{array}{l} d\left(n\right)=|\Phi \left(\text{n}\right)-\bar{\Phi \left(\text{n}\right)}{|}_{\text{F}}, \end{array}$$

(16) $$\begin{array}{l} \kappa \left(n\right)=\{\begin{array}{c} 1,\;d\left(n\right)\leq \sigma \\ 0,\;d\left(n\right)>\sigma \end{array}, \end{array}$$

Where Φ(**n**) is a 4 × 1 vector representing the relative phases, and $\bar{\Phi (n)}$ is the mean value of the previous Φ(**n**). The value of N was set to 50 in the experiments. *d*(*n*) is the Frobenius norm, which indicates the variance of the relative phases. σ is a threshold that was set empirically to 0.4. Based on the experiments we conducted, this value can indicate that a stable gait is formed. Thus, the ACI outputs modulating the CPGs are introduced as follows:


(17) $$\begin{array}{l} g_{i}\left(n\right)=\kappa \left(n\right)\xi \overset{4}{\underset{k=1}{\sum }}\left(\sin\left(o_{i}^{l}\left(n\right)-o_{i}^{k}\left(n\right)-\phi_{lk}\left(n\right)\right)\right),\;i=1,2, \end{array}$$

Where $o_{i}^{l}\left(n\right)$ and $o_{i}^{k}\left(n\right)$ are the outputs of the *i*th neurons in CPGs *l* and *k* (*l* and *k* = 1, 2, 3, 4). ϕ_*lk*_(*n*) is the relative phase of CPG *k*, with respect to CPG *l*. ξ is a communication gain, which we empirically set to 0.01.

By introducing *g*_*i*_(*n*) for CPG modulation, the new activations of CPG neurons are as follows:


(18) $$\begin{array}{l} a_{i}\left(n+1\right)=\overset{2}{\underset{j=1}{\sum }}w_{ij}o_{j}\left(n\right)-f_{i}\left(n\right)+g_{i}\left(n\right),\;i=1,2, \end{array}$$

Where *f*_*i*_(*n*) and *g*_*i*_(*n*) represent the modulation terms of the adaptive sensory feedback and neural couplings produced by the APC and ANC, respectively.

## 3. Experiments and results

Several experiments were conducted on Lilibot to assess the effectiveness of the proposed adaptive neural control (APNC-based control) (see Supplementary Figures S1, S2, Supplementary Table S1). First, the APNC-based control was evaluated in a real-time physical simulation (CoppeliaSim) for autonomously generating adaptive walking patterns at different CPG frequencies, update frequencies, terrain roughness, and robot conditions. We also compared the performance of the APNC-based control with two state-of-the art self-organized locomotion control methods (i.e., Tegotae-based control Kano et al., 2017; Owaki et al., 2017, 2021 and PR-based control Nomura et al., 2009; Aoi et al., 2011, 2012, 2021; Ambe et al., 2021). Similar to the setup of the proposed method, both methods also use GRFs to modulate the phase relationships of distributed (decoupled) CPGs to generate self-organized locomotion. Continuous GRFs are typically used to modulate CPG phases in the Tegotae method (also known as phase modulation Sun et al., 2021b). The PR method, on the other hand, uses discrete GRFs to periodically reset the CPG phases. The parameter setups of the three control methods are presented in Supplementary Tables S2, S3. Second, we examined the functions of the APC for quickly generating self-organized locomotion, following which we tested the effectiveness of the APNC for realizing robust and reusable self-organized locomotion in the real world. Finally, the maneuverability of the robot using the formed locomotion was demonstrated by changing the walking speed and direction under manual steering control in the real world. In the simulated robot, the GRFs were detected using force sensors in the legs, while in the real robot, they were calculated from motor current feedback in the knee joints (Sun et al., 2020).

### 3.1. Adaptability of the adaptive neural control

#### 3.1.1. Adaptability to different CPG frequencies

**Figure 4:**
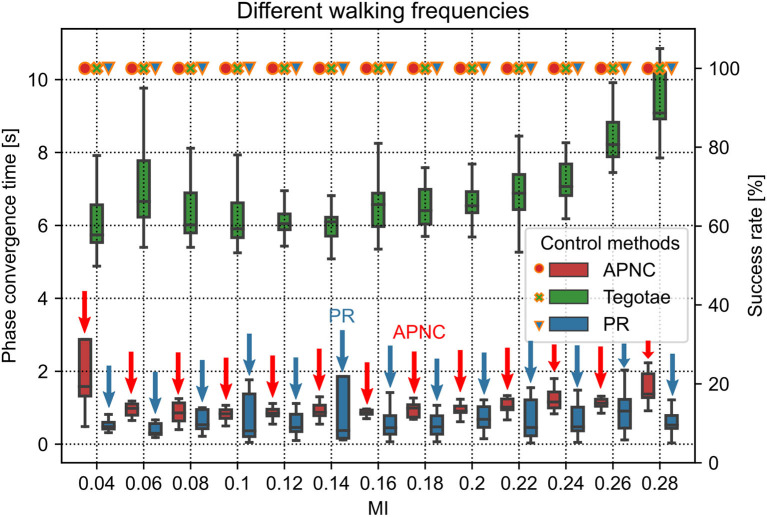
Phase convergence time (represented by the boxes along with the left axis) and success rate (represented by the marks along with the right axis) of the robot performing self-organized locomotion under three different control methods (APNC, Tegotae, and PR) and different *MI* values (corresponding to different walking frequencies). Small and large *MI* values indicate low and high walking frequencies, respectively. The success rate is defined as the ratio of successful phase convergences to total trials (i.e., 20). The red and blue arrows indicate the APNC and PR.

Legged robots and animals show adaptive walking patterns with respect to the changes of their walking speed (Hoyt and Taylor, 1981; Owaki et al., 2012; Owaki and Ishiguro, 2017; Fukui et al., 2019; Nirody, 2021). In the physical simulation, we experimented the APNC on Lilibot to evaluate its adaptive walking pattern generation (self-organized locomotion) under different *MI* values (i.e., different walking frequencies, see Equation 3). The CPG parameter *MI* determines the CPG output frequency and robot step frequency (*S*_*f*_), thereby regulating the robot's walking speed. There is an approximate relationship between them: *S*_*f*_ = 8.6 × *MI* + 0.5. Thirteen different *MI* values ranging from 0.04 to 0.28 (comparable to *S*_*f*_ from 0.8 to 2.9 Hz) were tested. The experiment was repeated 20 times for each *MI* value. Each trial ran for 45 s. The adaptability was evaluated using the CPG phase convergence time and duty factor (see Figure 4).

The CPG phase convergence time indicates the time required by the relative phases (ϕ_12_(*n*), ϕ_13_(*n*), ϕ_14_(*n*)) of the decoupled CPGs to converge to a suitable state (e.g., π, π, 0) from the initial state (0, 0, 0).^3^ The dynamic transitions of the relative phases can be seen in Supplementary Figure S3.

The CPG phase convergence time and success rate were explored at different walking frequencies for the three control methods (Figure 4). The APNC and PR-based control methods exhibited faster phase convergence than the Tegotae-based control method. The APNC-based control method employed an adaptive feedback gain to properly accelerate the effect of the continuous phase modulation; thus, it achieved faster phase convergence than the Tegotae-based control method, which employed a fixed predefined feedback gain. The PR-based control method used discrete GRF feedback to periodically reset the CPG phases, which also led to fast phase convergence (see Sun et al., 2021b for further comparative analysis of the continuous phase modulation and PR).

The duty factor is defined as the proportion of the stance phase to a gait cycle (swing and stance phases). It can quantify the walking patterns according to the foot-end movement states. The average duty factors of the four legs are interestingly proportional to the *MI* value (see Supplementary Figure S4). This is due to the APC mechanism. The frequency of the CPG oscillation increases as the *MI* value increases (Manoonpong et al., 2013). This also increases the walking or step frequency, and the higher the step frequency, the greater the GRF feedback. As a result, the actual GRF signals might be greater than the expected GRF signals; thereby, the adaptive gains are also increased to transmit the actual GRF signals for adapting/inhibiting the CPGs' activations. Thus, the stance phase can become longer than the swing phase (see the gait diagram^4^ in Supplementary Figure S5). When *MI* ≥ 0.24, the effect weakens, and the average duty factor tends to be stable at approximately 0.72.

#### 3.1.2. Adaptability to different controller update frequencies

**Figure 5:**
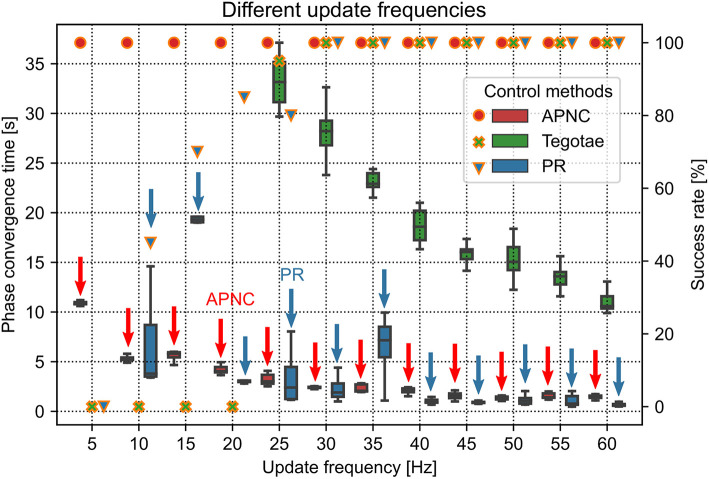
Phase convergence time (represented by the boxes along with the left axis) and success rate (represented by markers along with the right axis) of the robot performing self-organized locomotion under three different control methods (APNC, Tegotae, and PR) for various update frequencies of the robot system. Red and blue arrows indicate the APNC and PR.

The update frequency of the robot system is an important factor for self-organized locomotion generation. We conducted robot walking experiments using different update frequencies ranging from 5 Hz to 60 Hz to investigate the effect of update frequency on control performance (Figure 5). The robot motor system has a maximum update frequency of 60 Hz. For the experiments, we tested three control methods (APNC, Tegotae, and PR) and compared their phase convergence time and success rate. Each trial lasted 60 s and was repeated 20 times. The *MI* value defining the robot walking frequency was set to 0.08.

Figure 5 shows the success rate and phase convergence time of each control method for different update frequencies. In general, as the update frequency increased, the phase convergence time of the three control methods decreased. This is because the high update frequency had low delay of the sensory feedback to the CPG, thereby yielding the optimal CPG modulation. Only the APNC method successfully generated self-organized locomotion with 100% success rate for all the update frequencies. The Tegotae and PR methods achieved 100% success rate only when the frequency was higher than 25 Hz. Furthermore, owing to its adaptive sensory feedback gain (see Figure 2) that properly enhances the effect of GRF modulation in the CPG phase, the APNC method achieved phase convergence faster than the Tegotae method across all the frequencies. The stability analysis of the control system under different update frequencies is provided in Supplementary Figure S10.

#### 3.1.3. Adaptability to different terrain roughness

**Figure 6:**
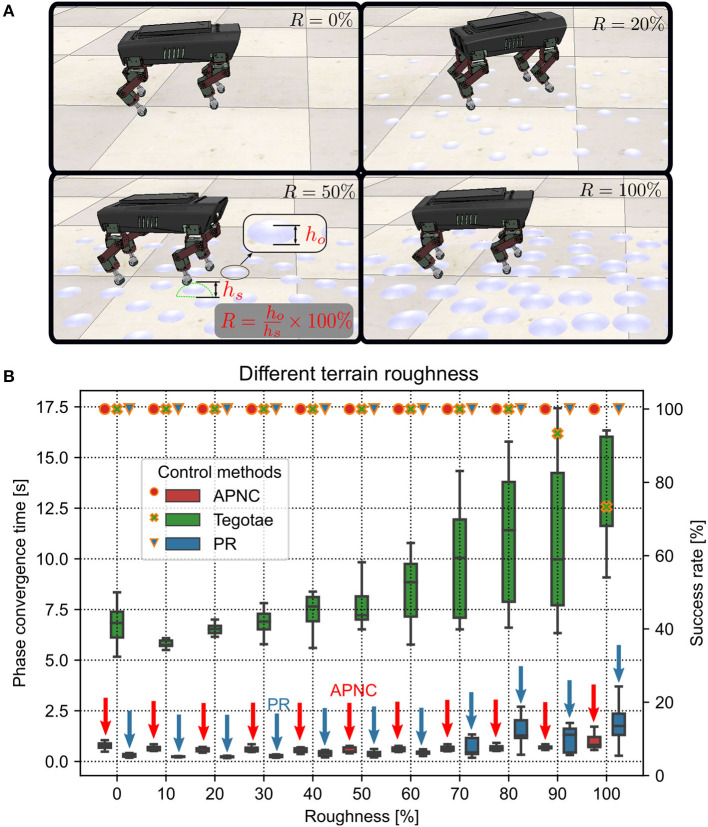
**(A)** Illustrations of four uneven terrain examples with different roughness (*R*) (0%, 20%, 50%, and 100%). The robot walking behavior related to the different terrain roughness conditions can be viewed at http://www.manoonpong.com/AdaptiveCommunications/video2.mp4. **(B)** Phase convergence time (represented by the boxes along with the left axis) and success rate (represented by marks along with the right axis) of the robot performing self-organized locomotion under the three different control methods (APNC, Tegotae, and PR) and various terrain roughness conditions from flat (*R* = 0%) to extremely rough terrains (*R* > 60%). The success rate was defined as the ratio of successful phase convergences to total trials (i.e., 20). Red and blue arrows indicate the APNC and PR. Note that the phase convergence time, indicating the efficiency of a controller enabling the robot to achieve self-organized locomotion, is used here as a measure to assess how fast a control system can learn or adapt. However, other measures, like speed and balance, can be also used to evaluate the robustness of rough terrain locomotion.

Adaptability to various uneven terrains (characterized by terrain roughness) is a critical property of adaptive interlimb coordination mechanisms for legged robots. To explore the adaptability of the APNC to uneven terrains, we set up different terrain conditions with many randomly distributed hemispherical obstacles with different diameters embedded in the ground. This was done to emulate varying terrain roughness conditions. The roughness (*R*) was defined as the percentage of the obstacle height (*h*_*o*_) to the robot step height (*h*_*s*_) (see Figure 6A). We tested the robot on 10 different terrain roughness setups ranging from 0% to 100% in the simulation. The experiment on each roughness setup was performed 20 times, and each trial was run for 45 s. The robot step height was empirically set to 0.01265 m in the experiment. Note that a higher step height will lead to unstable locomotion which requires additional posture balance control (Kimura et al., 2007).

The experiment results are presented in Figure 6B. As can be observed, the CPG phase adaptation required more time to converge with increasing roughness across all the methods. Moreover, the deviation of the phase convergence time also increased. When *R* was larger than 80%, the Tegotae was unable to form a gait in some trials (i.e., the success rates were less than 100%). This was because the CPG phase relationships could not converge to the desired state (see Supplementary Figure S6). The experiment results indicated that the APNC not only adapted to all the extents of terrain roughness, including relatively extremely uneven terrains (*R* > 60%), but also exhibited faster and more stable phase convergence than the Tegotae and PR at the extremely uneven terrains.

#### 3.1.4. Adaptability to different robot conditions

To validate the performance of the proposed control method in complex conditions, we tested the robot under four conditions: normal condition (C1) as a baseline, noisy feedback [GRF with noise (C2)], leg damage (C3), and carrying a payload (C4) (see Supplementary Table S5 for more details). The robot's walking performance was evaluated based on three common metrics: i) balance, ii) coordination, and iii) cost of transport (COT). The metrics are defined in the Supplementary Section S6 and Sun et al. (2021a). The walking trial was repeated 20 times for each condition and control.

**Figure 7:**
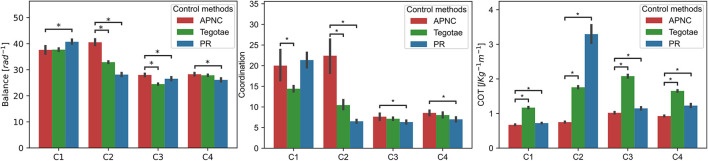
Comparison of the robot's walking performance under three different control methods (APNC, Tegotae, and PR) under four conditions (C1: normal condition, C2: noisy feedback, C3: leg damage, and C4: carrying payload (see Supplementary Table S5). To compare the performance of the control methods, a Mann-Whitney test was used. * denote significant differences with the following *p* ≤ 0.05. Note that the coordination metric is based on the duty factor. Thus, the low variance of the duty factor characterizes well-coordinated locomotion behavior (see also Supplementary Equations S4–S7).

**Figure 8:**
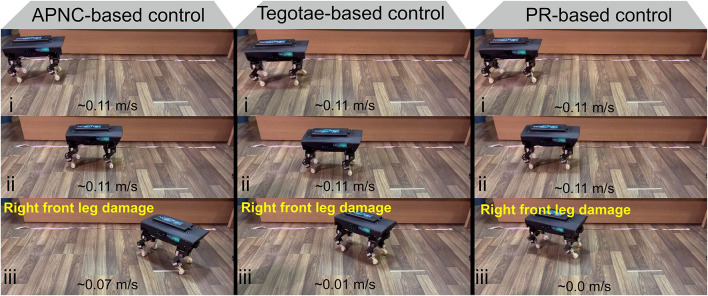
The three control methods were evaluated in a walking experiment conducted using the real robot with damaged leg. The APNC-based control enabled the robot to walk at the highest speed (0.07 m/s). A video clip of this experiment can be viewed at https://www.manoonpong.com/AdaptiveCommunications/video3.mp4.

The experiment results are depicted in Figure 7. While all the control methods (APNC, Tegotae, and PR) performed nearly equally well in the normal condition (C1), the APNC improved the robot's balance, coordination, and energy efficiency significantly, compared to the others in complex conditions (C2-C4). This is because, unlike those of the Tegotae and PR methods,^5^ the CPGs of the APNC-based control had adaptive neural connections that enabled the robot to effectively and robustly coordinate its leg movement even in complex conditions (C2-C4). In addition, a comparison of the three control methods using the real robot with a damaged leg is shown in Figure 8. As can be observed, the robot driven by the APNC-based control walked with the greatest speed.

### 3.2. Robust and reusable self-organized locomotion

**Figure 9:**
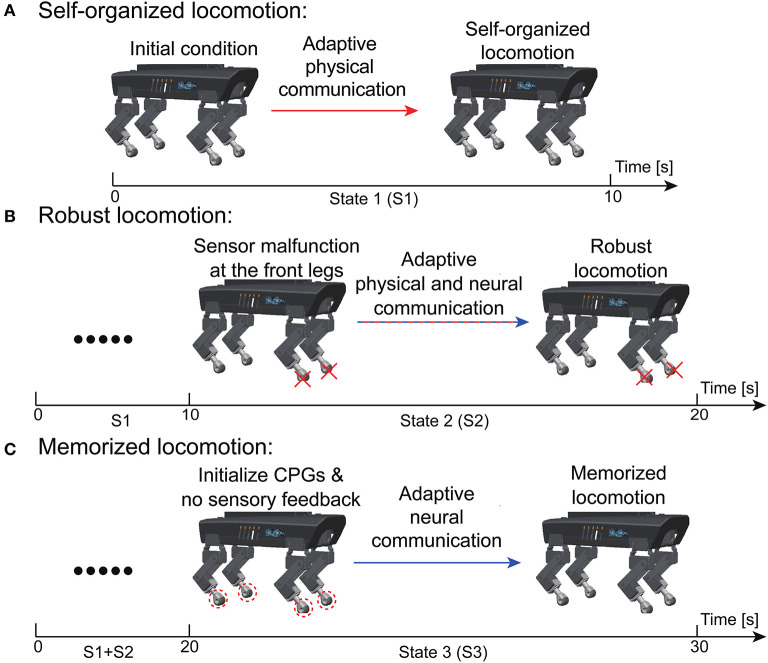
Experiment setup to evaluate the performance of the adaptive neural control (see text for details). **(A)** Self-organized locomotion. **(B)** Robust locomotion. **(C)** Memorized locomotion.

To systematically demonstrate the APNC-based adaptive neural control in the real world, a scenario consisting of three continuous states experienced by Lilibot was designed (see Figure 9). The states included self-organized locomotion generation from an initial condition (State 1, S1); sensory feedback malfunction (State 2, S2); and resetting to the initial condition (State 3, S3). In these situations, Lilibot, under the adaptive neural control (Figure 1), exhibited several locomotion properties, including (Figure 1A) self-organized locomotion in S1, Figure 1B robust locomotion in S2, and Figure 1C memorized or reusable locomotion in S3. To verify the functionalities of the ANC, S2 and S3 were performed twice, using the control with and without the ANC, for comparison purposes.

#### 3.2.1. Self-organized locomotion

**Figure 10:**
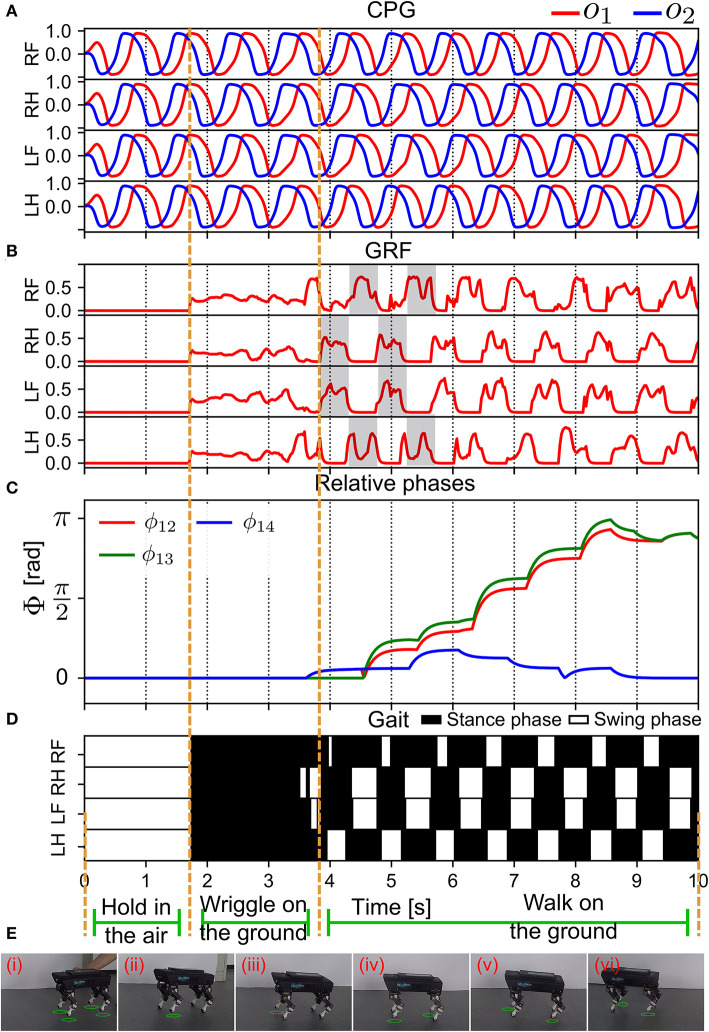
Real-time data of the self-organized locomotion generation (in S1). **(A)** The outputs of all the CPGs. **(B)** The GRF feedback to the CPGs. The GRF periodic pattern appeared after 4 s. **(C)** The relative phases between the CPG signals (see Equation 13). **(D)** Gait diagram. **(E)** Snapshots of Lilibot exhibiting self-organized locomotion. A green circle under a foot indicates that the foot was in a swing phase. Lilibot was suspended in midair in (i), and was placed on the ground in (ii), after which it began to form a gait from (iii) to (iv).

In the first state (S1), we suspended Lilibot midair and initialized all the CPGs (*MI* = 0.15) with the same parameters and phases. After 1.7 s, Lilibot was placed on the ground (see Figure 10). Consequently, the GRFs were activated to modulate the CPGs. The feedback gain (γ(*n*) in Equation 5) was also automatically adjusted *via* the DL (Equation 9) to obtain the proper feedback strength to effectively modulate the CPGs. The sensory feedback to each decoupled CPG was implemented by physical body dynamics (Figure 2). This indicates that the APC occurred among the CPGs.

After 3.8 s, the GRF signals exhibited a periodic pattern (see Figure 10B). Furthermore, the clear swing and stance phases, where the GRF exhibited no activation (swing) and high activation (stance), could be observed. This resulted in a stable, self-organized trot gait (Figure 10D). The relative phases acquired by the APR (Figure 3) converged at approximately 9 s (Figure 10C). The relative phases slowly converged (after approximately 5 s) because of the effect of a low-pass filter in the APR of each CPG control circuit. The relative phases indicated that the outputs of the CPG were in phase for the RF and LF legs and in anti-phase for the other legs. This experiment result demonstrated that the APNC enabled swift generation of self-organized locomotion (within 5 s) and effectively established the ANC on an even ground within 9 s. The snapshots of the experiment are shown in Figure 10E and a video clip of the experiment can be viewed at http://www.manoonpong.com/AdaptiveCommunications/video4.mp4.

After the self-organized locomotion emerged, the phase relationships (ϕ_*lk*_(*n*) in Equation 12) converged; thus, the physical communication gain (γ(*n*) in Equation 9) of the APC became small and the GRF feedback modulation (*f*_*i*_(*n*) in Equation 5) decreased, while the ANC was activated to induce neural couplings (*g*_*i*_(*n*) in Equation 17) among the CPGs, thereby storing the phase relationships (Supplementary Figure S7).

#### 3.2.2. Robust locomotion

**Figure 11:**
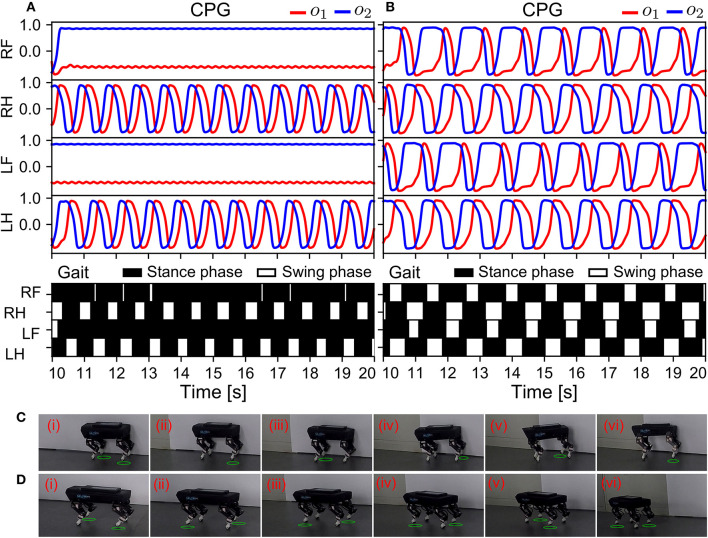
Real-time data of the robust locomotion and robot behavior negotiating a sensor malfunction (in S2). The experiment was conducted using the control without and with the ANC. The results are shown in **(A, B)**, respectively. **(C, D)** Snapshots of Lilibot under the adaptive neural control without and with the ANC, respectively. A green circle under a foot indicates the foot was in a swing phase.

In the second state (S2), after Lilibot performed self-organized locomotion, the ANC was activated to compensate for sensor malfunction (Figure 9B). Thus, in this state, there were two communication mechanisms (APC and ANC) working in parallel. To simulate possible sensor malfunctions arising from an unexpected impact, the sensory feedback of the front legs was set to a high constant value (i.e., 0.9). To comparatively examine the effect of the combination of both communications, the state was also tested without the ANC. The experiment results of the adaptive neural control without and with the ANC are shown in Figures 11A, B, respectively.

In Figure 11A, the outputs of the CPGs of the front legs became constant. This indicated that the two CPGs stopped oscillating, owing to the abnormal sensory feedback of the front legs, which was very high, at 0.9, inhibiting the CPGs through the physical communication. As shown in the gait diagram in Figure 11A, the front legs remained in the stance phase all the time because they could not periodically move. Conversely, in Figure 11B, all the CPGs continued to oscillate, even after the front legs encountered the same abnormal situation as in Figure 11A, because the ANC among the CPGs coupled them, enabling them to synchronize their activations. This compensated for the inhibitory effect caused by the sensor malfunction of the front legs. Consequently, the robot successfully maintained a trot gait. The robot's GRFs and displacement can be seen in Supplementary Figure S9. The experiment results of this state revealed that the APNC-based adaptive neural control successfully improved the robustness of the self-organized locomotion by compensating for sensor malfunction or damage. The snapshots of this experiment are shown in Figures 11C, D, and a video of the experiment can be viewed at http://www.manoonpong.com/AdaptiveCommunications/video5.mp4.

#### 3.2.3. Memorized locomotion

**Figure 12:**
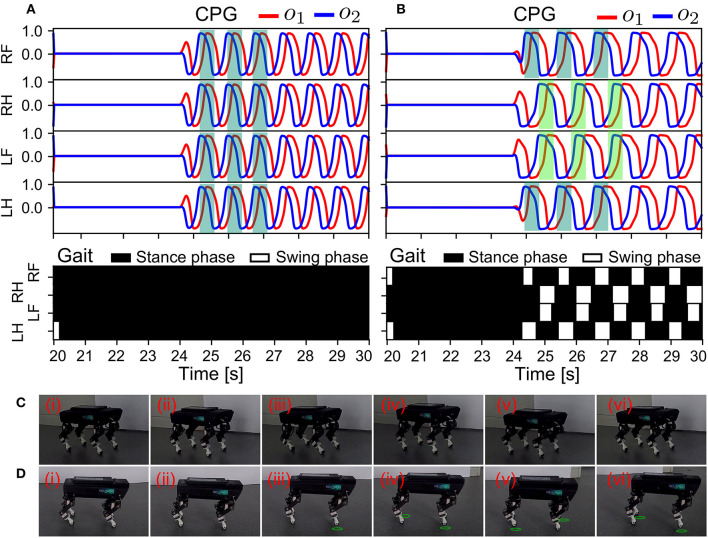
Real-time data of the memorized locomotion and robot behavior in response to the resetting of the control in S3. The experiment was conducted using the control without and with the ANC. The results are shown in **(A, B)**, respectively. The different colors indicate phase shifts among the CPGs in **(B)**. **(C, D)** Snapshots of Lilibot under the adaptive neural control without and with the ANC, respectively. A green circle under a foot indicates that the foot was in the swing phase.

In the last state (S3), we evaluated the effectiveness of the ANC for reusable locomotion or locomotion recovery. Therefore, we switched off the GRF feedback to the CPGs (i.e., sensory absence) such that there was no physical communication (see Figure 9C). Thus, only the ANC remained active. Furthermore, all the CPGs were initialized to prevent oscillation by setting *MI* (see Equation 3) to zero, thereby removing the formed patterns of the CPGs. S3 was performed under the adaptive neural control without and with the ANC. The results are shown in Figures 12A, B.

As shown in Figure 12, from 20 s to 24 s, the two cases yielded the same results during initialization. The CPGs stopped oscillating and the robot was motionless. At 24 s, the CPGs were reactivated to oscillate (we set *MI* of the CPGs back to 0.15). Subsequently, the behaviors of the robot were obviously different in both cases. In Figure 12A, although the CPGs generated commands to move the robot joints, the four legs moved in phase. The legs could not be lifted off the ground during the swing phase (see the gait diagram). Thus, the robot was rooted to the ground and unable to move forward. This was because without the ANC, the previously formed gait could not be recovered. Conversely, as shown in Figure 12B, the relative phases returned to the same values as observed in S1 (Figure 10). Accordingly, the trot gait was immediately recovered. This was attributed to the ANC, because the phases among the CPGs were stored in the couplings (i.e., motor memory; see Equation 17). The robot's GRFs and displacement can be seen in Supplementary Figure S8. The snapshots of this experiment are shown in Figures 12C, D, and a video clip of the experiment can be viewed at http://www.manoonpong.com/AdaptiveCommunications/video6.mp4.

### 3.3. Maneuverability of self-organized locomotion

**Figure 13:**
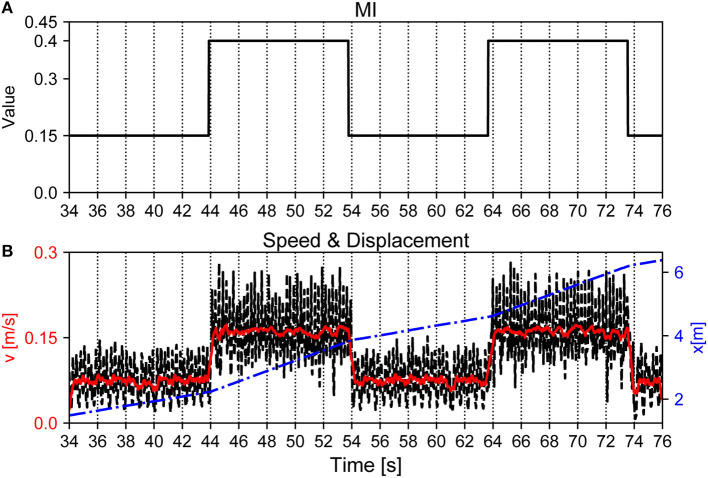
Walking speed of Lilibot adjusted by tuning *MI* of the CPGs. **(A)** Changes of *MI* of the CPGs. **(B)** Walking speed of the robot during the corresponding time. The black and red lines are the instantaneous and average speeds of the trunk with reference to the inertial frame, respectively. The blue line indicates the displacement of the robot.

Finally, we evaluated the maneuverability of Lilibot under the APNC-based adaptive neural control, where the walking speed and direction of the robot were controlled. In principle, the walking speed can be regulated by adjusting the *MI* value of the CPGs (Equation 3), the *a*_1,2_ values of the MNs (Equation 4), and the controller update frequency. A higher controller update frequency can result in a higher walking step frequency, higher *a*_1,2_ values can result in a larger stride length, and a higher *MI* value can result in a higher CPG oscillation frequency. As a consequence, all of these parameters can increase the robot walking speed. As an example, we demonstrated the walking speed control by setting the *MI* value of the CPGs (see Figure 13). We increased the *MI* value of the CPGs to 0.4 between 44–54 s and 64–74 s using a joystick; thereby leading to a faster walking speed. In the corresponding periods, the walking speed changed from approximately 0.08 m/s to 0.16 m/s. Once the lower *MI* value (e.g., 0.15) was set, the walking speed returned to the slower speed (0.08 m/s).

**Figure 14:**
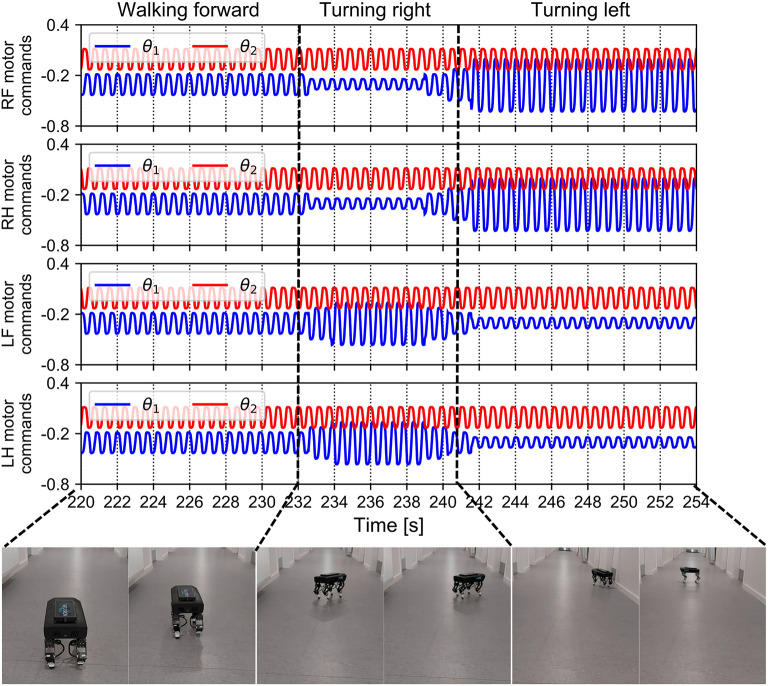
Motor commands fed to Lilibot and its walking direction under these commands. The four legs received periodic commands with the same amplitudes after the control converged and the robot walked forward. After approximately 232 s, the robot was made to turn right by setting the amplitudes of the left leg motor commands to be larger than normal and those of the right side became lower than normal. After 241 s, the setting of the amplitudes was reversed and the robot turned left.

The walking direction can be easily controlled by setting the magnitudes of the motor neuron outputs through the slope of the transfer function in the MNs (i.e., parameter *a*_*i*_ in Equation 4). The slope can be adjusted online in the proposed control *via* the ROS parameter server using the joystick. The results of the walking direction operation are presented in Figure 14. The aim of the walking direction regulation experiment was to set different slopes for the motor neurons (between the right and left sides of the robot). For instance, if the slopes of the right legs' motor neurons were steeper than those of the left legs' motor neurons, the robot turned left (see 242–254 s in Figure 14), and vice versa (see 232–241 s in Figure 14). A video clip of this continuous demonstration, including walking speed and direction regulation, can be viewed at http://www.manoonpong.com/AdaptiveCommunications/video7.mp4.

**Figure 15:**
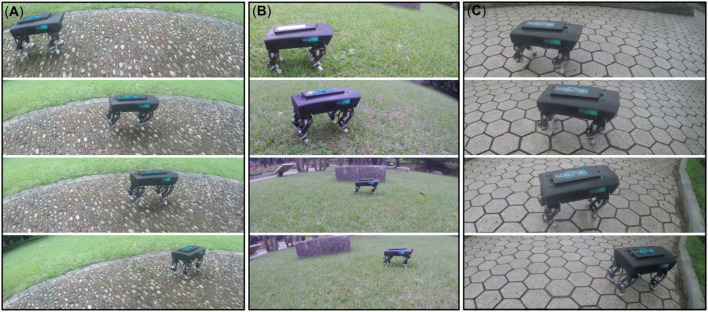
Outdoor demonstration on various terrain types. **(A)** Gravel. **(B)** Grass. **(C)** Pavement. The formed gait was then stored through the neural couplings. This enabled maneuverability, with the robot being able to stably change its walking speed and direction.

Additionally, we also demonstrated the performance of the adaptive neural control for self-organized locomotion and maneuverability of Lilibot on various types of outdoor terrains (e.g., gravel, grass, pavement, Figure 15). A video of this demonstration can be viewed at http://www.manoonpong.com/AdaptiveCommunications/video8.mp4.

## 4. Discussion

We proposed adaptive neural control based on the integration of the APC and ANC for robust and reusable self-organized locomotion of quadruped robots. The self-organized locomotion or automatic sensorimotor interlimb coordination (Aoi et al., 2017) was accomplished through the dynamic interactions among the decoupled neural CPG-based control circuits, sensory feedback, and the environment. The intralimb coordination of each leg was achieved through a local neural CPG-based control circuit that outputs two stable periodic signals. Both signals were applied to the hip and knee joints to generate proper foot movement (see Figure 2). Interlimb coordination was driven by four identical CPG-based control circuits that were coordinated and coupled by the APC and ANC. The APC built a communication channel through the GRF feedback to coordinate the CPG phase relationships and the ANC stored the formed CPG phase relationships as neural couplings between the CPG-based control circuits.

### 4.1. Aspect of the APC

In this study, the APC relied on the GRF feedback, which indirectly reflected the movement state of the legs owing to a mechanical connection between the legs and trunk. The mechanical connection and the interaction between the robot and the environment (see Equation 5) provided a physical communication channel among the CPGs through the GRF feedback. Specifically, when the legs moved on the ground, the GRF feedback inhibited the CPG activation using the feedback value (see Equation 5). This function induced phase differences among the identical CPGs because the GRFs of all the legs were not identical owing to noise, body movement dynamics, and non-perfect structural symmetry. Subsequently, the phase differences gradually converged because the physical communication produced a stable relationship among the CPGs through the GRFs. For example, when a leg (e.g., the RF leg) landed on the ground (*f*_1,2_ > 0 in Equation 5), the influence of the physical communication slowed the RF leg movement such that it remained in the stance phase slightly longer, and hence, 1) the leg provided additional time to wait for the other legs to enter their stance phase and 2) shared the load acting on the other legs to enable them to enter their swing phase. After the other legs entered the stance phase, thus supporting the trunk, the GRF of the RF leg began to decrease and gradually became zero, inducing the leg to enter a swing phase (*f*_1,2_ = 0 in Equation 5). Consequently, the effect gradually yielded stable phase shifts among the CPGs, thereby generating self-organized locomotion.

In the dynamic interaction induced by the APC, there were three vital factors to consider: 1) the differences among the GRF feedback of the CPGs, which triggered phase shifts among identical CPGs, regardless of the fact that they had the same initialization; 2) the sensory feedback gain (γ in Equation 5) that defined the ideal inhibitory strength applied to the CPGs, thus impacting the effectiveness of the convergence in the self-organized locomotion process significantly. When the inhibition was too strong, the CPGs stopped oscillating. On the other hand, the convergence process was very slow when the inhibition was too weak; and 3) the functionality of the sensory feedback that provided an appropriate modulation for the CPG phases to generate proper interlimb coordination. We elaborate on the three points subsequently.

#### 4.1.1. Differences among the GRF feedback

The legs received the same motion commands if their CPGs were initialized using the same parameters. Thus, in an ideal situation, they would perform the same movements in response to the same commands. Accordingly, in the ideal condition, the sensors on the legs would acquire the same sensory information (e.g., GRFs) and pass it on to the CPGs. This resulted in the same modulation for all the CPG activations, such that the legs would move in the same phase forever. Therefore, in this ideal case, the physical communication makes it impossible for the robot to form a gait. However, the actual GRFs of all legs were not completely identical, even when the legs moved in the same phase. This is because of the existence of some non-ideal factors such as sensory noise, joint movement trace error, measurement error, and imperfect structural symmetry. These factors cause slight differences among GRFs, thus creating initial phase shifts among the CPGs. Subsequently, the slight phase shifts increase rapidly and converge to values that represent a particular gait when using sensory feedback modulation (see Equation 5).

#### 4.1.2. Sensory feedback gain

The adaptive feedback gain mechanism employed in this study augmented the differences among the GRF feedback to accelerate gait formation. This is because of the ability of the DL to derive the optimal feedback gain for each leg, thus enabling the GRF to appropriately influence the CPG. In Owaki et al. (2012), Owaki et al. proposed a novel approach using four decoupled phase oscillators serving as CPGs with local-only GRF feedback to generate self-organized locomotion in a quadruped robot. They discussed the phase dynamics of the sensory-modulated CPG and indicated that the CPG had two states, namely, the oscillatory and excitatory states. The autonomous switch from one state to the other plays an essential role in the generation of adaptive interlimb coordination. However, the sensory feedback gain was predefined in their work, although it has a significant effect on the convergence time of self-organized locomotion. Therefore, we induced an adaptive sensory feedback gain realized by the DL (see Figure 2A). The DL adjusted the gain depending on the error between the actual and expected GRFs such that the actual leg movement matched the expected one with greater precision. The DL with fast and slow learners quickly adapted the feedback gain online toward achieving rapidly self-organized locomotion generation (i.e., within 5 s see Figure 10). The error between the actual and expected GRFs utilized by the DL was limited to positive values (Equation 6). Consequently, the feedback gain was non-negative (γ ≥ 0) and had a positive value (γ > 0) only when the corresponding foot remained in the actual stance phase; thus, the DL was active when the robot interacted with the ground. This feature prevented the legs' swing phase from affecting the retention of the DL such that the adaptive sensory feedback modulation provided a rapid reaction to the stance phase movement.

#### 4.1.3. Functionality of the sensory feedback

The automatic sensorimotor coordination was significantly influenced by the functionality of the sensory feedback, which determined the walking patterns. Over the years, various forms of sensory information have been exploited to modulate CPGs for adaptive gait generation and transition. For instance, Fukuhara et al. investigated a combination of the support and propulsion force as sensory feedback to adjust the CPG phase, through which they demonstrated not only self-organized locomotion generation, but also gait transitions on a quadruped robot (Fukuhara et al., 2018). Fukui et al. (2019) modulated the CPG phase using vestibular feedback, which enabled a quadruped robot to achieve autonomous gait transition and galloping, even when the CPGs had predefined and fixed weak connections. In addition to the use of multiple sensory feedback, Aoi et al. (2010) studied the effect of the stiffness of the backbone joint on gait transitions under CPG-based control. They achieved gait transitions by changing the waist joint stiffness. Similar biological examples have been observed in mammals (e.g., dogs Schilling and Carrier, 2010, cheetahs Hildebrand, 1959 and horses Hildebrand, 1959) that exhibit distinctive spine movements when they use different gaits.

As a matter of fact, the backbone joint significantly affects the functionality of the physical communication because its stiffness influences the dynamic interactions among the legs. In summary, these results support the argument that the functionality of sensory feedback to the CPGs not only plays a key role in interlimb coordination generation, but also affects the gait selection. In this study, we used Lilibot, a robot with a rigid trunk that provided a relatively fixed transmission over GRFs (i.e., a support force) regardless of changes in the walking speed and direction changes. Thus, in future work, we plan to implement multiple sensory feedback modulation and incorporate a backbone joint with adaptive stiffness to extend the flexibility of the physical communication channel and thereby enable the robot to perform adaptive gait transitions.

Owing to its high dependence on sensory feedback, there are two drawbacks associated with using the APC for interlimb coordination generation. First, once a sensor malfunction occurs, the robot cannot sustain its stable self-organized locomotion. Second, the robot cannot memorize the formed gait for later recovery. However, biological findings have demonstrated that animals have the capability to maintain locomotion even when they encounter unexpected situations (Graham, 1977) such as sensory damage or leg amputation. A general neurophysiological fact is that the neural couplings between neural circuits form a basis for memory. An evidence from Giovanni et al. demonstrated that neural circuits and their couplings support imitation learning of hand actions (Buccino et al., 2004). Many biologically inspired control approaches employed for locomotion generation utilize neural couplings or connections to predefine movement behaviors for legged robots (Cruse et al., 1998; Kimura et al., 2007; Ajallooeian et al., 2013; Fukuoka and Kimura, 2014; Liu et al., 2018).

### 4.2. Aspect of the ANC

Neural couplings endue robots with more stable and reusable interlimb coordination. In contrast to the traditional predefined neural couplings, we implemented adaptive neural couplings, which were automatically formed online to obtain the ANC among the distributed CPGs. Consequently, we integrated the neural communication and physical communication mechanisms to achieve rapid self-organized locomotion that was characterized by 1) robustness against sensor damage, as well as 2) reusability of a formed gait for movement recovery.

#### 4.2.1. Robust self-organized locomotion

The generated self-organized locomotion was robust against sensory feedback damage. In our experiments, we studied a sensor malfunction case in which the GRFs to the front legs took on a high constant value to simulate an unexpected collision triggering sensor failure in the feet (see S2 in Figure 9). The abnormal GRF signals could not be used to distinguish the movement state (stance or swing phase) and strongly inhibited the CPGs continuously, as a result of which the CPGs stopped oscillating (Figure 11). However, the control with the additional ANC was able to synchronize and enforce the oscillation of the CPGs, even if some of them are inhibited heavily by abnormal sensory feedback. This is because the activations of the CPGs were controlled by the CPGs themselves through the ANC (see Equation 17). Therefore, the control using a combination of the APC and ANC enabled a legged robot to exhibit not only rapidly self-organized locomotion generation but also robust locomotion against sensor malfunction or absence. This result indicates that the ANC mechanism plays an important role in supplementing physical communication to generate stable movement in a legged robot. Furthermore, in contrast to some existing CPG coupling approaches using phase oscillators (Collins and Richmond, 1994; Aoi et al., 2010), we achieved neural communication based on the abstract version of biological neurons (i.e., the neuron model in the SO(2) CPG). This biological plausibility tends to support the claim that adaptive neural couplings (e.g., synaptic adaptation) play a crucial role regarding interlimb coordination after a limb injury (e.g., leg loss Cully et al., 2015 or amputation Dasgupta et al., 2015).

#### 4.2.2. Reusable self-organized locomotion

Another important effect attributed to the ANC is the reusability of the formed locomotion even when the CPGs are reinitialized. The robot exhibiting this behavior demonstrated that the proposed adaptive neural control can memorize the stable movement formed in a self-organized manner. This feature was derived from the inherent properties of the adaptive neural couplings, which stored the stable phase relationships among the CPGs. Motor learning has increasingly attracted researchers aiming to develop adaptive movement in robotics (Reinkensmeyer et al., 2004). This study illustrates a means to convert the adaptive movement formed online (using physical communication) into an adaptive neural circuit network (see Figure 1B). It further provides insight into the underlying mechanism of motor memory regarding how to encode and store information in the neural system for movement recovery in robots.

### 4.3. Combination of the APC and ANC

Based on the real-time GRF feedback, the APC modulates and forms a walking pattern, which the ANC stores and recalls. Their gains (γ(*n*) in Equation 9 and ξ in Equation 17) determine their respective contributions in shaping the walking pattern. In the maneuverability experiment, the neural communication gain (ξ) was set to a relatively high value (i.e., 0.01) compared to the physical communication gain (γ), which automatically converged toward zero (γ(*n*) < 0.01, see the DL output in Figure 2B) after the neural communication was active. Thus, the recall function of the ANC was stronger than the modulation function of the APC. Although the changing walking speed and directions produced different GRF feedback to the CPGs, the ANC strongly stabilized the generated walking pattern. The balance between the two parameters defined the interplay between the APC and ANC. Similar to the terminology in RL (Hwangbo et al., 2019; Jones et al., 2020; Thor et al., 2020), the roles of the APC and ANC represented the exploration (learning process) and exploitation (recall process) in our locomotion learning, respectively.

The proposed adaptive neural control with the APC and ANC enabled a robot to quickly generate adaptive gaits within 9 s on even terrain (Figure 10), 20 s on simulated rough terrain (Figure 6B), and 25 s on outdoor terrain (e.g., gravel, grass, and pavement), unlike conventional ML-based control that typically requires long training and learning processes. For instance, Ishige et al. presented a combination of CPG-based control with an episode-based RL for locomotion generation. Its training process required approximately a day (Ishige et al., 2019). Hwangbo et al. (2019) proposed an RL-based method for training a neural network policy in simulation and transferred it to a quadruped robot ANYmal. This approach also required a long training process (approximately 4 h). To avoid long training sessions resulting from the structural complexity of neural networks (Hwangbo et al., 2019), Thor and Manoonpong recently presented a novel control framework that translates CPG signals into desired joint motor commands for robot locomotion *via* a radial basis function network with a simplified structure and black-box optimization (Thor et al., 2020). However, it still required several training sessions (approximately 13 min for normal walking). In addition, Juang et al. presented a multi-objective evolution method based on an ant colony algorithm to learn a recurrent neural network for generating the gait of a legged robot. This method required a few billion of trials for training in simulation, following which the trained model was transferred to a real robot (Juang and Yeh, 2018).

A comparison of the proposed adaptive neural control with related state-of-art methods (Buchli and Ijspeert, 2008; Aoi et al., 2012; Owaki et al., 2012; Barikhan et al., 2014; Fukuhara et al., 2018; Miguel-Blanco and Manoonpong, 2020) for fast self-organized locomotion generation (i.e., obtaining a gait in less than a minute and without robot kinematics, environmental models, and predefined interlimb coordination) reveals that the proposed adaptive neural control method can achieve self-organized locomotion on not only even terrain (typically shown by the others Buchli and Ijspeert, 2008; Aoi et al., 2012; Owaki et al., 2012; Barikhan et al., 2014; Fukuhara et al., 2018; Miguel-Blanco and Manoonpong, 2020) but also uneven terrain. Furthermore, it provides motor memory through the ANC for gait recovery and robust locomotion to deal with a sensor malfunction. It also enables spontaneous variations in the walking speed and direction for robot maneuverability (Supplementary Table S6). It is worth noting that, while the proposed control system has been shown to be effective in the study, it does not address adaptive intralimb coordination, which controls the robot foot trajectory and has a significant impact on the robot's balance on complex uneven terrain and slopes (Sun et al., 2021a).

### 4.4. Limitations

The proposed control method has two limitations of the intralimb coordination that determines the robot foot trajectory and significantly influences the robot's balance on uneven terrains. First, the relative phase between the outputs of a CPG was a fixed value ≈ π/2 that defined the intralimb coordination between the hip and knee joints of a leg. In addition, the hip and knee joint movement amplitudes and offsets, which influence the step length and body posture (Wang et al., 2018; Sun et al., 2020; Saputra et al., 2022), were also predefined with fixed parameter values. These two points limited the adaptation of the intralimb coordination of the robot, thus hindering its ability to effectively handle complex terrains (e.g., slopes Sun et al., 2021a) and negotiate a high obstacle (Sun et al., 2018). Biological studies have revealed that adaptive interlimb and intralimb coordination as well as posture control depends on the integration of CPGs, reflexes, and muscle mechanisms (Aoi et al., 2017; Saputra et al., 2022). Thus, in the future, we will integrate multiple reflexes (e.g., vestibular/posture reflexes Kimura et al., 2007 and spinal reflex Saputra et al., 2022) and muscle models (Xiong et al., 2015) to realize a more advanced adaptive intralimb coordination and study its integration with the proposed adaptive interlimb coordination.

## 5. Conclusion

We developed adaptive neural control by integrating the APC and ANC. The experiment results on Lilibot indicate that the combination of the APC and ANC can enable more robust and reusable locomotion. It further confirms that the combination plays an essential role in reliable interlimb coordination generation in biological, as well as artificial systems.^6^ The main advantages of the proposed approach over existing locomotion control approaches, such as classic engineering techniques (Raibert et al., 2008; Hutter et al., 2016; Semini et al., 2017; Bledt et al., 2018) and ML (e.g., RL Nakamura et al., 2007; Heess et al., 2017; Hwangbo et al., 2019; Ishige et al., 2019; Jones et al., 2020, ant-colony optimization Juang and Yeh, 2018, intelligent trial and error Cully et al., 2015, and black-box optimization Thor et al., 2020) are as follows:

- It does not require robot kinematics, environmental models, and predefined interlimb coordination (i.e., the interlimb coordination was achieved in a self-organized manner),
- It does not require numerous attempts and long convergence time (i.e., we were able to quickly generate robust and reusable self-organized locomotion within a few seconds (i.e., 9 s and 25 s for even and uneven terrains, respectively).

These features make the proposed approach powerful and generic for developing robust and reusable self-organized locomotion for legged robots. The control based on the proposed approach is modular and developed with generic interfaces. It is flexible and offers the possibility of integrating it with other control strategies such as balance control through a reflex mechanism (Kimura et al., 2007) and navigation. Moreover, the control can be extended to various types of legged robots, such as hexapod, and octopod, because the adaptive neural control is organized by distributed identical local control circuits, and the relationships among the local control circuits are formed in an adaptive manner. This will enable the adaptive neural control to be used as a generic control algorithm for various legged systems in the future. Limitations of the proposed method are that some parameters were empirically set up, such as α and ρ of the FM, as well as phase shift, amplitudes, and offsets of intralimb coordination. The parameter values lead to the expected GRF with a predefined ideal shape and predefined foot trajectory, thereby reducing the adaptability of the control to some extent. In the future, we will further investigate optimizing the control method parameters by combined with reinforcement learning.

## Data availability statement

The raw data supporting the conclusions of this article will be made available by the authors, without undue reservation.

## Author contributions

TS implemented the control methods, analyzed the data, and wrote the original manuscript. ZD supervised the study. PM fully supervised this study (including the research idea, experimental design, and experimental data analysis) and wrote the manuscript. All authors contributed to the article and approved the submitted version.

## Footnotes

^1^Refer to the cattle video at http://www.manoonpong.com/AdaptiveCommunications/cattle.mp4 and a documentary: amazing nature: run to survive by Free High-Quality Documentaries at https://youtu.be/ibzAZyO5wmM4.

^2^SO(2) is a specific type of two-neuron network with recurrent connections. The outputs of the neurons are sine wave-like patterns and have a phase shift of π/2 created using specific parameter configurations.

^3^Note that, a suitable state of CPG relative phases is determined from the ideal state that quadruped robots perform a typical trot gait where the front and hind legs on opposite sides move together. However, the converged relative phases might be slightly changed around this ideal state depending on the situations of the robot and environment as well as their interactions. Thus, for simplicity here, we use the ideal state (π, π, 0) as our reference state to measure phase convergence time.

^4^A video showing robot walking gaits at different *MI* values can be seen at http://www.manoonpong.com/AdaptiveCommunications/video1.mp4.

^5^It is worth noting that the Tegotae and PR methods rely solely on physical communication to form a robot gait. They do not include adaptive neural communication, and therefore cannot store or memorize a generated gait (i.e., no motor memory). Consequently, removing the GRF feedback may cause their locomotion systems to become unstable, as continuous GRF feedback is required to maintain the stability of the generated gait. In contrast, the proposed locomotion system successfully memorized and reused the generated gait *via* the ANC.

^6^ http://www.manoonpong.com/AdaptiveCommunications/video9.mp4

## References

[B1] Ajallooeian M. Pouya S. Sproewitz A. Ijspeert A. J. (2013). “Central pattern generators augmented with virtual model control for quadruped rough terrain locomotion,” in 2013 IEEE International Conference on Robotics and Automation (Karlsruhe: IEEE), 3321–3328.

[B2] Ambe Y. Aoi S. Tsuchiya K. Matsuno F. (2021). Generation of direct-, retrograde-, and source-wave gaits in multi-legged locomotion in a decentralized manner via embodied sensorimotor interaction. Front. Neural Circ. 15, 706064. 10.3389/fncir.2021.706064 34552472 PMC8450536

[B3] Aoi S. Amano T. Fujiki S. Senda K. Tsuchiya K. (2021). Fast and slow adaptations of interlimb coordination via reflex and learning during split-belt treadmill walking of a quadruped robot. Front. Robot. AI 8, 697612. 10.3389/frobt.2021.697612 34422913 PMC8378330

[B4] Aoi S. Egi Y. Sugimoto R. Yamashita T. Fujiki S. Tsuchiya K. (2012). Functional roles of phase resetting in the gait transition of a biped robot from quadrupedal to bipedal locomotion. IEEE Trans. Robot. 28, 1244–1259. 10.1109/TRO.2012.2205489

[B5] Aoi S. Fujiki S. Yamashita T. Kohda T. Senda K. Tsuchiya K. (2011). “Generation of adaptive splitbelt treadmill walking by a biped robot using nonlinear oscillators with phase resetting,” in 2011 IEEE/RSJ International Conference on Intelligent Robots and Systems (San Francisco, CA: IEEE), 2274–2279.

[B6] Aoi S. Manoonpong P. Ambe Y. Matsuno F. Wörgötter F. (2017). Adaptive control strategies for interlimb coordination in legged robots: a review. Front. Neurorobot. 11, 39. 10.3389/fnbot.2017.00039 28878645 PMC5572352

[B7] Aoi S. Yamashita T. Ichikawa A. Tsuchiya K. (2010). “Hysteresis in gait transition induced by changing waist joint stiffness of a quadruped robot driven by nonlinear oscillators with phase resetting,” in 2010 IEEE/RSJ International Conference on Intelligent Robots and Systems (Taipei: IEEE), 1915–1920.

[B8] Barikhan S. S. Wörgötter F. Manoonpong P. (2014). “Multiple decoupled cpgs with local sensory feedback for adaptive locomotion behaviors of bio-inspired walking robots.,” in From Animals to Animats 13 (Cham: Springer International Publishing), 65–75.

[B9] Bledt G. Powell M. J. Katz B. Di Carlo J. Wensing P. M. Kim S. (2018). “MIT cheetah 3: Design and control of a robust, dynamic quadruped robot,” in 2018 IEEE/RSJ International Conference on Intelligent Robots and Systems (Madrid: IEEE), 2245–2252.

[B10] Buccino G. Vogt S. Ritzl A. Fink G. R. Zilles K. Freund H.-J.. (2004). Neural circuits underlying imitation learning of hand actions: an event-related fmri study. Neuron 42, 323–334. 10.1016/S0896-6273(04)00181-3 15091346

[B11] Buchli J. Ijspeert A. J. (2008). Self-organized adaptive legged locomotion in a compliant quadruped robot. Auton. Robots. 25, 331–347. 10.1007/s10514-008-9099-2

[B12] Collins J. J. Richmond S. A. (1994). Hard-wired central pattern generators for quadrupedal locomotion. Biol. Cybern. 71, 375–385. 10.1007/BF00198915

[B13] Cruse H. Kindermann T. Schumm M. Dean J. Schmitz J. (1998). Walknet-a biologically inspired network to control six-legged walking. Neural Networks 11, 1435–1447. 10.1016/S0893-6080(98)00067-7 12662760

[B14] Cully A. Clune J. Tarapore D. Mouret J.-B. (2015). Robots that can adapt like animals. Nature 521, 503–507. 10.1038/nature14422 26017452

[B15] Dallmann C. J. Hoinville T. Dürr V. Schmitz J. (2017). A load-based mechanism for inter-leg coordination in insects. Proc. R. Soc. B Biol. Sci. 284, 20171755. 10.1098/rspb.2017.1755 29187626 PMC5740276

[B16] Dasgupta S. Goldschmidt D. Wörgötter F. Manoonpong P. (2015). Distributed recurrent neural forward models with synaptic adaptation and cpg-based control for complex behaviors of walking robots. Front. Neurorobot. 9, 10. 10.3389/fnbot.2015.00010 26441629 PMC4585172

[B17] Dickinson M. H. (2000). How animals move: An integrative view. Science 288, 100–106. 10.1126/science.288.5463.100 10753108

[B18] Fukuhara A. Owaki D. Kano T. Kobayashi R. Ishiguro A. (2018). Spontaneous gait transition to high-speed galloping by reconciliation between body support and propulsion. Adv. Robot. 32, 794–808. 10.1080/01691864.2018.1501277

[B19] Fukui T. Fujisawa H. Otaka K. Fukuoka Y. (2019). Autonomous gait transition and galloping over unperceived obstacles of a quadruped robot with cpg modulated by vestibular feedback. Rob. Auton. Syst. 111, 1–19. 10.1016/j.robot.2018.10.002

[B20] Fukuoka Y. Kimura H. (2014). Dynamic locomotion of a biomorphic quadruped 'tekken' robot using various gaits: walk, trot,free-gait and bound. Appl. Bionics Biomech. 6, 63–71. 10.1155/2009/743713

[B21] Graham D. (1977). The effect of amputation and leg restraint on the free walking coordination of the stick insectcarausius morosus. J. Compar. Physiol. 116, 91–116. 10.1007/BF00605519

[B22] Grillner S. Zangger P. (1984). The effect of dorsal root transection on the efferent motor pattern in the cat's hindlimb during locomotion. Acta Physiol. Scand. 120, 393–405. 10.1111/j.1748-1716.1984.tb07400.x 6741575

[B23] Heess N. Dhruva T. B. Sriram S. Lemmon J. Merel J. Wayne G.. (2017). Emergence of locomotion behaviours in rich environments. arXiv [Preprint]. arXiv: 1707.02286. Available online at: https://arxiv.org/abs/1707.02286

[B24] Hildebrand M. (1959). Motions of the running cheetah and horse. J. Mammal. 40, 481–495. 10.2307/137626518854295

[B25] Hoyt D. F. Taylor C. R. (1981). Gait and the energetics of locomotion in horses. Nature 292, 239–240. 10.1038/292239a0

[B26] Hülse M. Wischmann S. Manoonpong P. von Twickel A. Pasemann F. (2007). “Dynamical systems in the sensorimotor loop: on the interrelation between internal and external mechanisms of evolved robot behavior,” in 50 Years of Artificial Intelligence (Springer), 186–195.

[B27] Hutter M. Gehring C. Jud D. Lauber A. Bellicoso C. D. Tsounis V.. (2016). “Anymal-a highly mobile and dynamic quadrupedal robot,” in 2016 IEEE/RSJ International Conference on Intelligent Robots and Systems (IROS) (Daejeon: IEEE), 38–44.

[B28] Hwangbo J. Lee J. Dosovitskiy A. Bellicoso D. Tsounis V. Koltun V.. (2019). Learning agile and dynamic motor skills for legged robots. Sci. Robot. 4, aau5872. 10.1126/scirobotics.aau5872 33137755

[B29] Ijspeert A. J. (2008). Central pattern generators for locomotion control in animals and robots: a review. Neural Networks 21, 642–653. 10.1016/j.neunet.2008.03.014 18555958

[B30] Ijspeert A. J. Crespi A. Ryczko D. Cabelguen J.-M. (2007). From swimming to walking with a salamander robot driven by a spinal cord model. Science 315, 1416–1420. 10.1126/science.1138353 17347441

[B31] Ishige M. Umedachi T. Taniguchi T. Kawahara Y. (2019). Exploring behaviors of caterpillar-like soft robots with a central pattern generator-based controller and reinforcement learning. Soft Robot. 6, 579–594. 10.1089/soro.2018.0126 31107172 PMC6786347

[B32] Jones W. Blum T. Yoshida K. (2020). “Adaptive slope locomotion with deep reinforcement learning,” in 2020 IEEE/SICE International Symposium on System Integration (SII) (Honolulu, HI: IEEE), 546–550.

[B33] Juang C. Yeh Y. (2018). Multiobjective evolution of biped robot gaits using advanced continuous ant-colony optimized recurrent neural networks. IEEE Trans. Cybern. 48, 1910–1922. 10.1109/TCYB.2017.2718037 28682271

[B34] Kano T. Yoshizawa R. Ishiguro A. (2017). Tegotae-based decentralised control scheme for autonomous gait transition of snake-like robots. Bioinspirat. Biomimet. 12, 046009. 10.1088/1748-3190/aa7725 28581439

[B35] Kimura H. Fukuoka Y. Cohen A. H. (2007). Adaptive dynamic walking of a quadruped robot on natural ground based on biological concepts. Int. J. Rob. Res. 26, 475–490. 10.1177/0278364907078089

[B36] Kullander K. Butt S. J. Lebret J. M. Lundfald L. Restrepo C. E. Rydström A.. (2003). Role of epha4 and ephrinb3 in local neuronal circuits that control walking. Science 299, 1889–1892. 10.1126/science.1079641 12649481

[B37] Liu C. Li X. Zhang C. Chen Q. (2018). Multi-layered cpg for adaptive walking of quadruped robots. J. Bionic. Eng. 15, 341–355. 10.1007/s42235-018-0026-8

[B38] Lodi M. Shilnikov A. L. Storace M. (2020). Design principles for central pattern generators with preset rhythms. IEEE Trans. Neural Networks Learn. Syst. 31, 3658–3669. 10.1109/TNNLS.2019.2945637 31722491

[B39] Lundberg A. (1979). Multisensory control of spinal reflex pathways. Prog. Brain Res. 50, 11. 10.1016/S0079-6123(08)60803-1 121776

[B40] MacKay-Lyons M. (2002). Central pattern generation of locomotion: a review of the evidence. Phys. Ther. 82, 69–83. 10.1093/ptj/82.1.69 11784280

[B41] Manoonpong P. Parlitz U. Wörgötter F. (2013). Neural control and adaptive neural forward models for insect-like, energy-efficient, and adaptable locomotion of walking machines. Front. Neural Circ. 7, 12. 10.3389/fncir.2013.00012 23408775 PMC3570936

[B42] Marder E. Bucher D. (2001). Central pattern generators and the control of rhythmic movements. Curr. Biol. 11, R9NR-R996. 10.1016/S0960-9822(01)00581-4 11728329

[B43] Miguel-Blanco A. Manoonpong P. (2020). General distributed neural control and sensory adaptation for self-organized locomotion and fast adaptation to damage of walking robots. Front. Neural Circ. 14, 46. 10.3389/fncir.2020.00046 32973461 PMC7461994

[B44] Nakamura Y. Mori T. aki Sato M. Ishii S. (2007). Reinforcement learning for a biped robot based on a cpg-actor-critic method. Neural Networks 20, 723–735. 10.1016/j.neunet.2007.01.002 17412559

[B45] Nirody J. A. (2021). Universal features in panarthropod inter-limb coordination during forward walking. Integrat. Compar. Biol. 61, 710–722. 10.1093/icb/icab097 34043783 PMC8427173

[B46] Nomura T. Kawa K. Suzuki Y. Nakanishi M. Yamasaki T. (2009). Dynamic stability and phase resetting during biped gait. Chaos 19, 026103. 10.1063/1.3138725 19566263

[B47] Owaki D. Goda M. Miyazawa S. Ishiguro A. (2017). A minimal model describing hexapedal interlimb coordination: The tegotae-based approach. Front. Neurorobot. 11, 29. 10.3389/fnbot.2017.00029 28649197 PMC5465294

[B48] Owaki D. Horikiri S.-,y. Nishii J. Ishiguro A. (2021). Tegotae-based control produces adaptive inter-and intra-limb coordination in bipedal walking. Front. Neurorobot. 15, 47. 10.3389/fnbot.2021.629595 34054453 PMC8149599

[B49] Owaki D. Ishiguro A. (2017). A quadruped robot exhibiting spontaneous gait transitions from walking to trotting to galloping. Sci. Rep. 7, 277. 10.1038/s41598-017-00348-9 28325917 PMC5428244

[B50] Owaki D. Kano T. Nagasawa K. Tero A. Ishiguro A. (2012). Simple robot suggests physical interlimb communication is essential for quadruped walking. J. R. Soc. Interface 10, 20120669. 10.1098/rsif.2012.0669 23097501 PMC3565797

[B51] Pasemann F. Hild M. Zahedi K. (2003). “So (2)-networks as neural oscillators,” in International Work-Conference on Artificial Neural Networks (Berlin: Springer), 144–151. 10.1007/3-540-44868-3_19

[B52] Raibert M. Blankespoor K. Nelson G. Playter R. (2008). Bigdog, the rough-terrain quadruped robot. IFAC Proc. 41, 10822–10825. 10.3182/20080706-5-KR-1001.01833

[B53] Reinkensmeyer D. J. Emken J. L. Cramer S. C. (2004). Robotics, motor learning, and neurologic recovery. Ann. Rev. Biomed. Eng. 6, 497–525. 10.1146/annurev.bioeng.6.040803.140223 15255778

[B54] Saputra A. A. Botzheim J. Ijspeert A. J. Kubota N. (2022). Combining reflexes and external sensory information in a neuromusculoskeletal model to control a quadruped robot. IEEE Trans. Cybern. 52, 7981–7994. 10.1109/TCYB.2021.3052253 33635813

[B55] Schilling N. Carrier D. R. (2010). Function of the epaxial muscles in walking, trotting and galloping dogs: implications for the evolution of epaxial muscle function in tetrapods. J. Exp. Biol. 213, 1490–1502. 10.1242/jeb.039487 20400634

[B56] Semini C. Barasuol V. Goldsmith J. Frigerio M. Focchi M. Gao Y.. (2017). Design of the hydraulically actuated, torque-controlled quadruped robot hyq2max. IEEE/ASME Trans. Mechatron. 22, 635–646. 10.1109/TMECH.2016.2616284

[B57] Smith M. A. Ghazizadeh A. Shadmehr R. (2006). Interacting adaptive processes with different timescales underlie short-term motor learning. PLoS Biol. 4, e179. 10.1371/journal.pbio.0040179 16700627 PMC1463025

[B58] Steingrube S. Timme M. Wörgötter F. Manoonpong P. (2010). Self-organized adaptation of a simple neural circuit enables complex robot behaviour. Nat. Phys. 6, 224–230. 10.1038/nphys1508

[B59] Sun T. Dai Z. Manoonpong P. (2021a). Distributed-force-feedback-based reflex with online learning for adaptive quadruped motor control. Neural Networks 142, 410–427. 10.1016/j.neunet.2021.06.001 34139657

[B60] Sun T. Shao D. Dai Z. Manoonpong P. (2018). “Adaptive neural control for self-organized locomotion and obstacle negotiation of quadruped robots,” in 2018 27th IEEE International Symposium on Robot and Human Interactive Communication (Nanjing: IEEE), 1081–1086.

[B61] Sun T. Xiong X. Dai Z. Manoonpong P. (2020). Small-sized reconfigurable quadruped robot with multiple sensory feedback for studying adaptive and versatile behaviors. Front. Neurorobot. 14, 14. 10.3389/fnbot.2020.00014 32174822 PMC7054281

[B62] Sun T. Xiong X. Dai Z. Owaki D. Manoonpong P. (2021b). A comparative study of adaptive interlimb coordination mechanisms for self-organized robot locomotion. Front. Robot. AI 8, 86. 10.3389/frobt.2021.638684 33912596 PMC8072274

[B63] Thor M. Kulvicius T. Manoonpong P. (2020). Generic neural locomotion control framework for legged robots. IEEE Trans. Neural Networks Learn. Syst. 32, 4013–4025. 10.1109/TNNLS.2020.3016523 32833657

[B64] Tran D. T. Koo I. M. Lee Y. H. Moon H. Park S. Koo J. C.. (2014). Central pattern generator based reflexive control of quadruped walking robots using a recurrent neural network. Robot. Auton. Syst. 62, 1497–1516. 10.1016/j.robot.2014.05.011

[B65] Wang Y. Xue X. Chen B. (2018). Matsuoka's cpg with desired rhythmic signals for adaptive walking of humanoid robots. IEEE Trans. Cybern. 50, 613–626. 10.1109/TCYB.2018.2870145 30307884

[B66] Wark B. Lundstrom B. N. Fairhall A. (2007). Sensory adaptation. Curr. Opin. Neurobiol. 17, 423–429. 10.1016/j.conb.2007.07.001 17714934 PMC2084204

[B67] Wolpaw J. R. (2010). What can the spinal cord teach us about learning and memory? The Neuroscientist 16, 532–549. 10.1177/1073858410368314 20889964

[B68] Xiong X. Wörgötter F. Manoonpong P. (2015). Adaptive and energy efficient walking in a hexapod robot under neuromechanical control and sensorimotor learning. IEEE Trans. Cybern. 46, 2521–2534. 10.1109/TCYB.2015.2479237 26441437

[B69] Yu J. Tan M. Chen J. Zhang J. (2014). A survey on cpg-inspired control models and system implementation. IEEE Trans. Neural Networks Learn. Syst. 25, 441–456. 10.1109/TNNLS.2013.2280596 24807442

[B70] Zeng Y. Li J. Yang S. Ren E. (2018). A bio-inspired control strategy for locomotion of a quadruped robot. Appl. Sci. 8, 56. 10.3390/app8010056

