# Supplementary material for: Robust and reusable self-organized locomotion of legged robots under adaptive physical and neural communications
Source: Front Neural Circuits. 2023 Mar 31;17:1111285. doi: 10.3389/fncir.2023.1111285 (PMC10102392; doi:10.3389/fncir.2023.1111285)
Supplement: Supplementary file 1 [file Data_Sheet_1.pdf]

Supplementary Material

1 QUADRUPED ROBOT: LILIBOT

To test the performance of the proposed adaptive neural control in a physical system, a small-sized and open source quadruped robot platform (Lilibot[1]) with a flexible control framework was employed (Figure S1). The real Lilibot and its simulated model in a physical robot simulator (CoppeliaSim (V-REP) (Rohmer et al., 2013)) are controlled by the adaptive neural control through the Robot Operation System (ROS) interfaces. In this software framework (Figure S1(B)), various values of the simulated robot can be monitored and the ROS parameter server can easily adjust the parameters of the control. Therefore, the simulated robot can be used to first test the adaptive neural control, then directly transfer it to the real robot without any modification. The real Lilibot and control framework are described in the following sections.

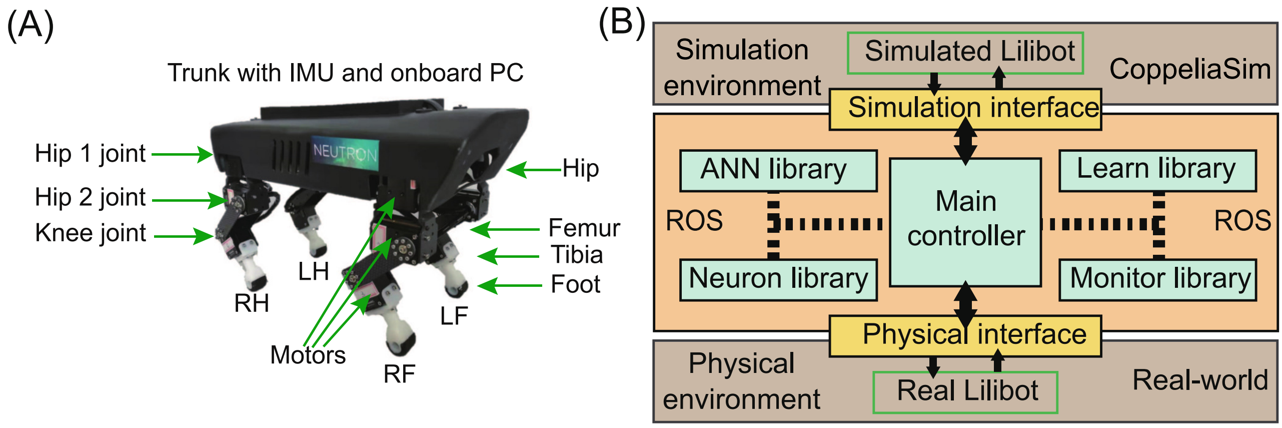

Figure S1: Details of the experimental quadruped robot (Lilibot). (A) Structural specifications of Lilibot. It has four identical legs, which are the right front (RF), right hind (RH), left front (LF), and left hind (LH) legs. Each leg has three active joints driven by a Dynamixel smart servo motor XM430-W350-R. The trunk carries an onboard PC and an inertial measurement unit (IMU) sensor. (B) Software framework for organizing codes to implement the proposed control algorithm.

Lilibot is designed with four identical light-weight legs assembled with an all-elbow configuration (Figure S1(A)). The legs are referred to as the RF, RH, LF, and LH legs. Each leg consists of three links, namely the hip, femur, and tibia, and has three active joints (hip 1 joint, hip 2 joint, and knee joint), which are driven by smart servo motors (4.2 Nm, XM430-W350-R from ROBOTIS). The smart servo motors can monitor the joint movement positions, velocities, motor voltages, and motor currents. The four knee joint motor currents are used to estimate the four legs' GRFs (Sun et al., 2020). The tibia link of each leg is connected to a foot that is covered by rubber material to create optimal friction. The links of the leg are constructed using 3D printing, and some of the parts are made of carbon fiber to provide high–strength, lightweight legs. The four legs are attached to a rigid trunk that carries an inertial measurement unit (IMU), an onboard PC, as well as a Li-ion battery (14.8 V, 4 Ah).

[1] https://gitlab.com/neutron-nuaa/lilibot

The type of the IMU is JY901 from Wit-motion[2]. The IMU module integrates a high-precision gyroscope, an accelerometer, a geomagnetic sensor, and a high-performance microprocessor. It utilizes a dynamic Kalman filter algorithm for sensory signal processing. The onboard PC is NUC7i7DNBE with 8GB DDR3 RAM from Intel. It has a core™i7-8650U processor (8M Cache, up to 4.20 GHz). The battery can supply the real Lilibot as a compact mobile platform to run for more than an hour. The sensors on the real robot are described in Table S1. Lilibot weighs approximately 2.5 kg. Its length, width, and height are 30 cm, 17.5 cm, and 20 cm, respectively, when standing.

Table S1. All sensors and amount of sensory feedback of Lilibot

Sensors	Sensory signals
An IMU on robot trunk (JY901 from Wit-motion)	Robot trunk angular velocity and acceleration
Contactless absolute encoders at each motor (AS5045 from AMS)	Joint positions and joint velocities
Integrated torque/current measurement at each motor (XM430-W350-R)	Joint current and torque, and estimated ground reaction force (GRF)

The framework of the adaptive neural control is implemented on the main controller, which can access all the modular and generic libraries (Figure S1(B)), such as artificial neural network (ANN) and neuron libraries for creating CPG models, a learning library for online learning and tuning the control parameters, and a monitoring library for monitoring the control operation. The communications between the control and the simulated and real robots are realized through the simulation and physical interfaces using ROS topics.

2 ROBOT CONTROL SETUP

2.1 Experiment platform setup

The experiments were implemented on both a simulated and real Lilibot, both of which can be controlled by the same adaptive neural control through ROS topics (motor topic and sensor topic, Figure S2). The parameter values of the control, such as the *MI* value of SO(2) CPG neurons for adjusting CPG frequency and the $a_{1,2}$ values of MN neurons for adjusting walking step length, are stored in the ROS parameter server. These parameter values can be regulated online using a joystick. The adaptive neural control is open-source, which can be accessed at `https://gitlab.com/neutron-nuaa/apnc`.

2.2 Control parameter setups

The adaptive neural control has several modules, which are formulated by discrete-time equations. The time step size of all the equations is determined by the update frequency of the control. The update frequencies of the control system were 40 Hz and 60 Hz in the real and simulation robot experiments, respectively. These update frequencies were fast enough for our real-time robot control. The parameter values of the modules can be seen in Table S2. The modules' parameter values were initialized as follows: 1) the parameter values of the CPGs and MNs were set such as to obtain proper intralimb coordination for Lilibot; 2) the FM parameter values were set such as to obtain the expected GRFs; 3) the DL parameter values were selected under the constraint that $B_f > B_s$, $A_f < A_s$; 4) the ANC parameter values were set empirically. It was not necessary to adapt the parameters of the DL to specific situations (Smith et al., 2006).

Tegotae-based control (Owaki et al., 2017; Kano et al., 2017; Owaki et al., 2021) and phase resetting (PR)-based control (Nomura et al., 2009; Aoi et al., 2010, 2012, 2021, 2011) were also employed to

[2] http://wiki.wit-motion.com/english/lib/exe/fetch.php?media=module:wt901:docs:jy901usermanualv4.pdf

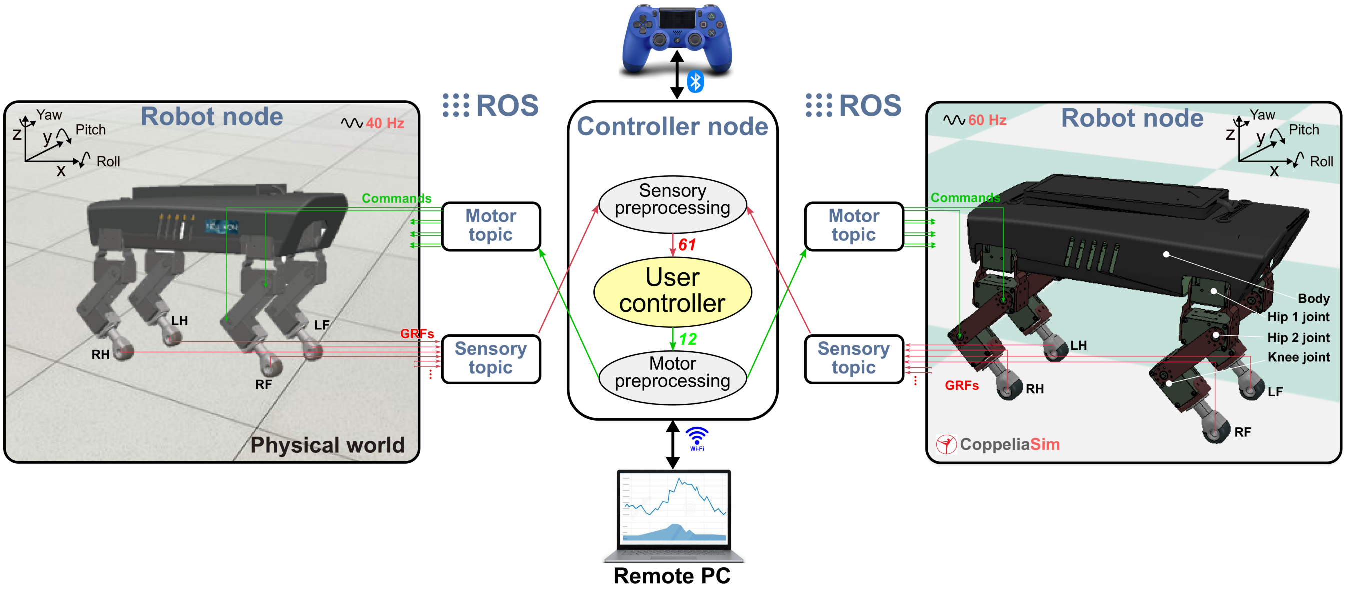

Figure S2: Framework of the adaptive neural control implemented on both the simulated and real Lilibots. The update frequencies of the control for the simulated and real robot experiments were 60 Hz and 40 Hz, respectively.

control Lilibot to comparatively evaluate the proposed adaptive neural control (APNC-based control). The mathematical expression of the Tegotae-based control and PR-based control can be seen in Sun et al. (2021). In this work, the parameter setups of the Tegotae-based and PR-based control methods are shown in Table S3. These values were empirically chosen to maximize the performance of the control in all the experiment trials.

Table S2. Parameter setup of the adaptive neural control

Modules	Symbols	Initial values	Description
CPGs	*MI*	0.08	Modulatory input of SO(2) CPG neurons (Equation (3) in the main manuscript). It determines the CPG frequency.
	$o_{1,2}$	0.01	Small initial outputs of the SO(2) CPG neurons to initially trigger the CPG oscillation (Equation (1) in the main manuscript).
MNs	$a_{1,2}$	0.1	The slopes of the linear transformation of the MNs (Equation (4) in the main manuscript). It regulates the walking step length.
	$b_{1,2}$	-0.2	The intercepts of the linear transformation of the MNs (Equation (4) in the main manuscript). It regulates the joint movement offsets.
APC	$\gamma(n)$	0.0	Adaptive physical communication gain modulated online by the DL (Equation (5) and Equation (9) in the main manuscript).
FM	α	0.9	Scaling factor of the expected GRF amplitude (Equation (10) in the main manuscript).
	ρ	0.99	Fine-tuning the duty factor of the expected GRF (Equation (10) in the main manuscript).
DL	A_f	0.57	Retention rate of the fast learner (Equation (7) in the main manuscript).
	B_f	0.002	Learning rate of the fast learner (Equation (7) in the main manuscript).
	A_s	0.99	Retention rate of the slow learner (Equation (8) in the main manuscript).
	B_s	0.0002	Learning rate of the slow learner (Equation (8) in the main manuscript).
ANC	N	50 (empirical)	The number of samples for calculating average relative phases (Equation (14) in the main manuscript).
	σ	0.4 (empirical)	The threshold to activate the ANC (Equation (16) in the main manuscript).
	ξ	0.01 (empirical)	The neural communication gain (Equation (17) in the main manuscript).
The update frequency of the control is 60 Hz in simulation and 40 Hz in real robot experiments			

Table S3. Parameter setup of the Tegotae-based and PR-based control

Control methods	Parameters	Value
Tegotae-based control	Sensory feedback gain	0.03
	CPG *MI*	0.08
	CPG $o_{1,2}$	0.01
PR-based control	GRF threshold factor	0.4
	CPG *MI*	0.08
	CPG $o_{1,2}$	0.01

3 DATA SOURCE OF FIGURES IN MANUSCRIPT

The data sources of all the figures in the manuscripts are listed in Table S4.

Table S4. Data source of the figures in the manuscript

Data sources	Figures
Data from the simulated robot	Figure 4, Figure 5, Figure 6 (B), Figure 7, Figure S3, Figure S4 Figure S5, Figure S6, Figure S7, and Figure S10
Data from the real robot	Figure 10, Figure 11, Figure 12, Figure 13, Figure 14 Figure S8, and Figure S9

4 VISUALIZATION OF SOME EXPERIMENTAL DATA

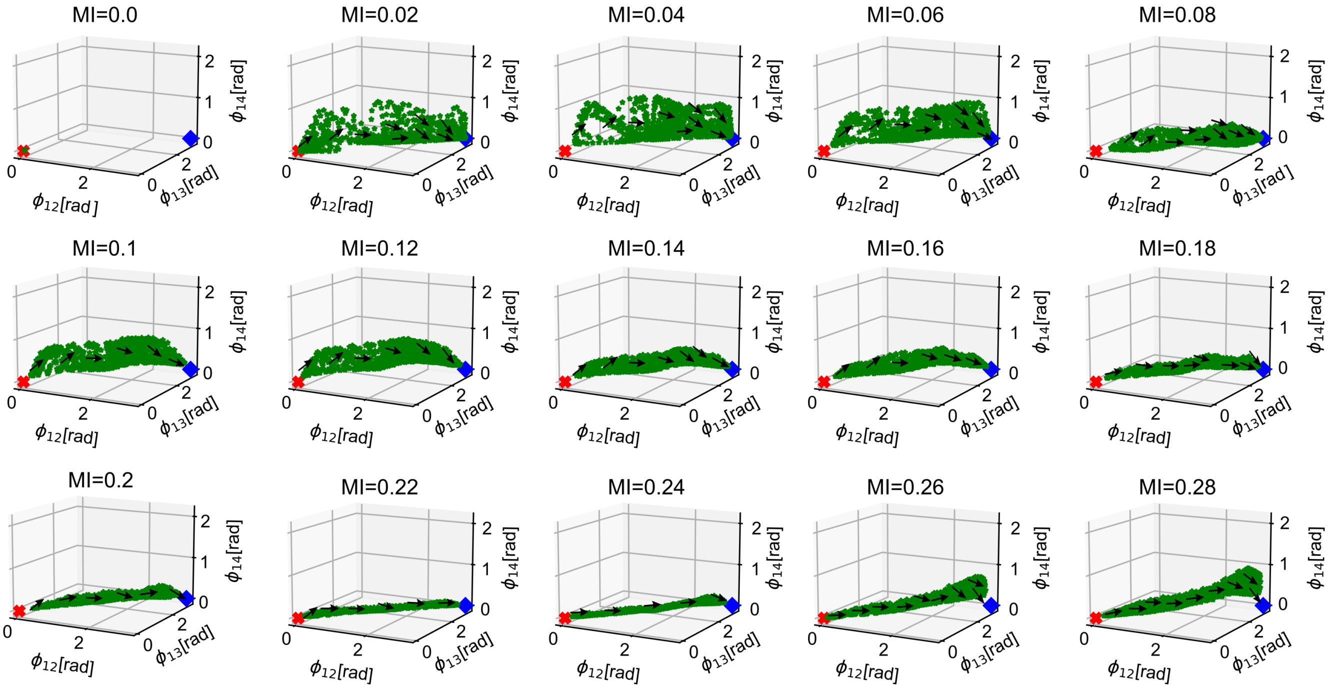

Figure S3: Phase portrait of the decoupled CPGs modulated by the APC under different *MI* values. The *MI* value affected the CPG frequency, thereby controlling the robot's walking speed. The red point (0,0,0) denotes the initial state and the blue point (π, π,0) denotes the desired state that represents the robot walking with a trot-like gait. The green points indicate the transitions of the CPG phase state during the process of the self-organized locomotion generation. We repeated the experiment 20 times for each *MI* value.

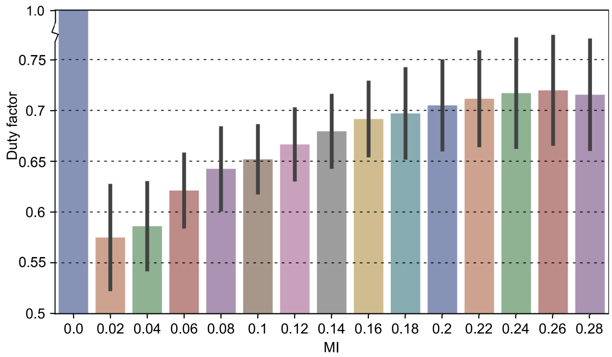

Figure S4: Average duty factors of the robot walking under the different *MI* values. A video showing the robot's walking behavior is at `http://www.manoonpong.com/AdaptiveCommunications/video1.mp4`.

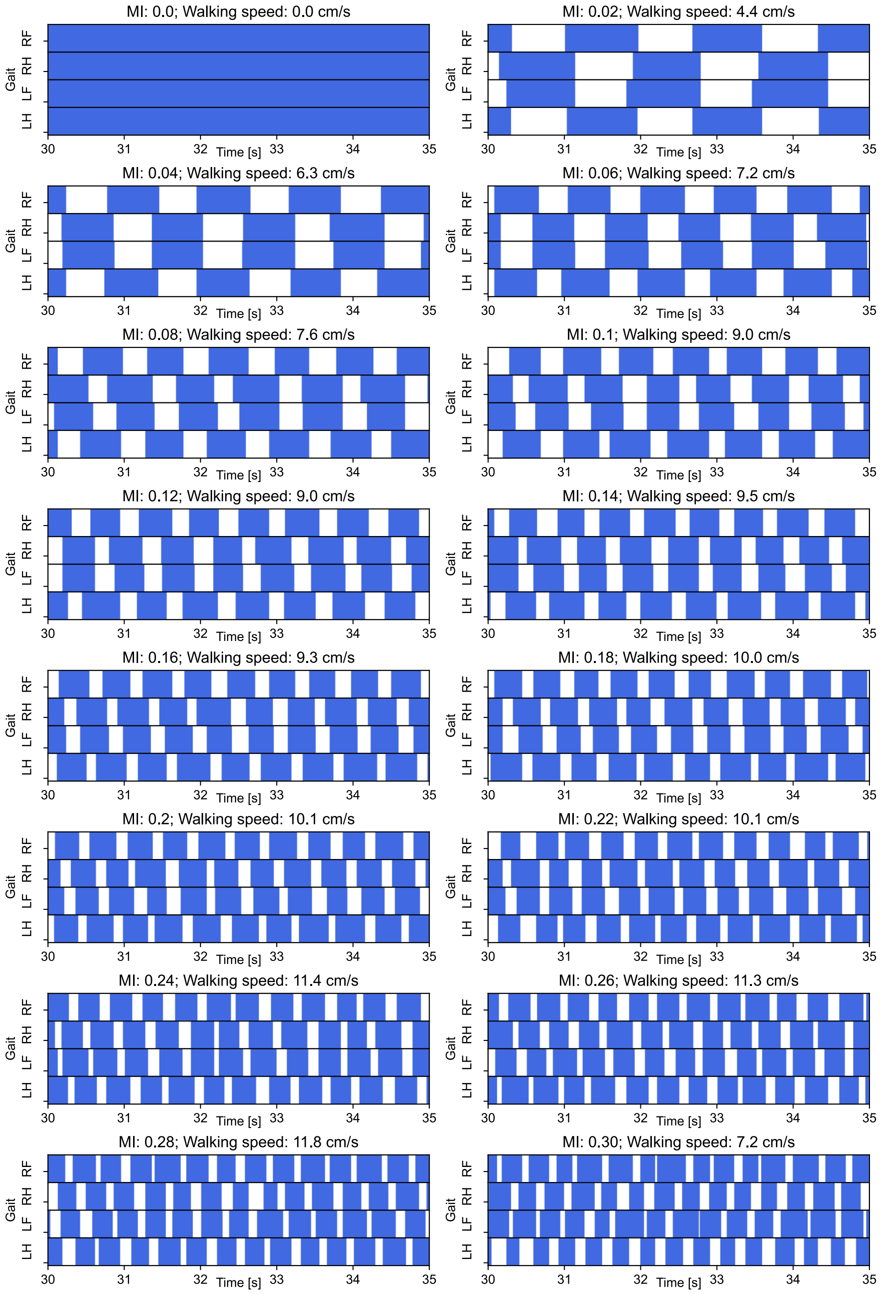

Figure S5: Gait diagrams of the robot walking under different speeds (different MI values). The blue and white regions represent the stance and swing phases, respectively. RF, RH, LF, and LH indicates the right front, right hind, left front, and left hind legs, respectively. Different gaits can be observed, including a trot gait where the RF and LH swing and the RH and LF were at the stance phase (MI=0.02,. . . ,0.16), as well as a modified trot gait where the RF, RH, LF, and LH were slightly shifted ($MI > 0.18$).

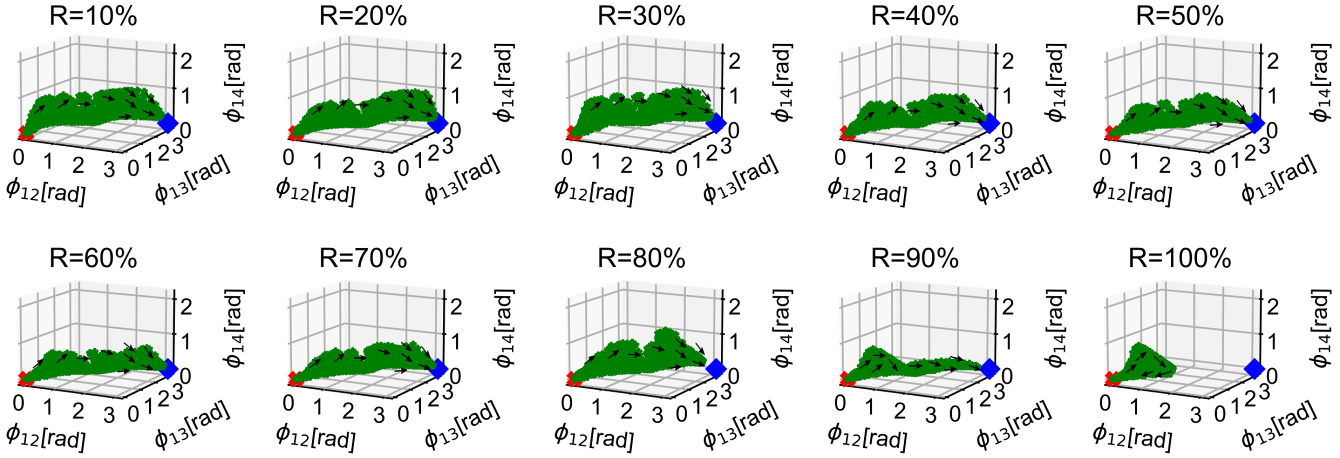

Figure S6: Phase portrait of the decoupled CPGs modulated in the Tegotae mechanism under varying terrain roughness (R). When $R \geq 80\%$, the state variables of the dynamical system (represented by the green points) were unable to completely converge to the desired state (i.e., (π, π, 0) blue point) from the initial state ((0, 0, 0) red point) in all the tests. In contrast, the dynamical system successfully converged to a state around the desired state when $R < 80\%$. In these cases, the robot performed a trot-like gait. We repeated the experiment 20 times for each roughness condition. The *MI* value defining the walking frequency was set to 0.08.

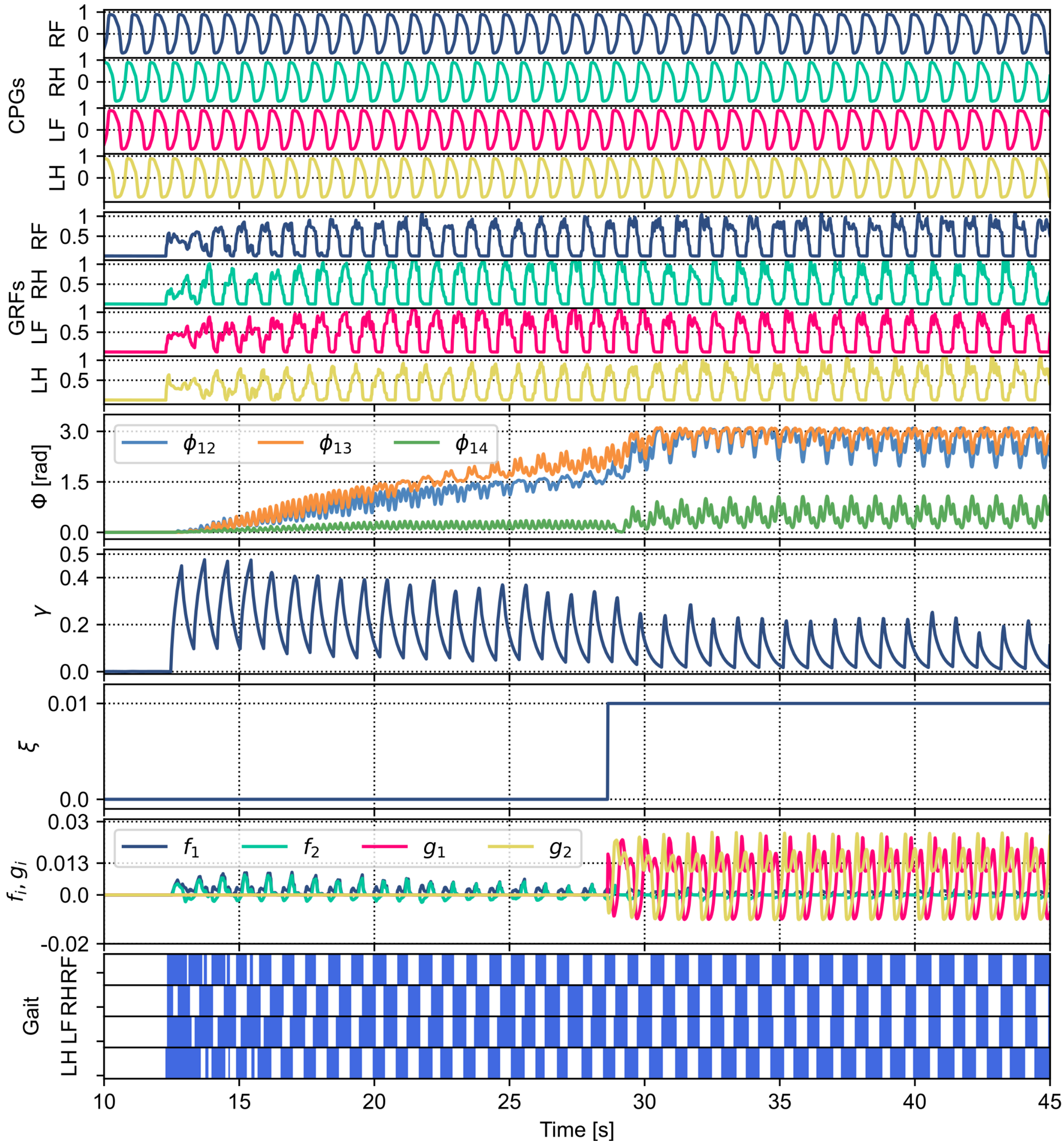

Figure S7: Real-time data of the self-organized locomotion of Lilibot controlled by the APNC. After the robot generated self-organized locomotion, the CPG phase relationships (ϕ_{lk}) converged, the GRF feedback modulation ($f_i(n)$ in Equation (5)) decreased owing to the physical communication gain ($\gamma(n)$ in Equation (9)) became small, and the ANC with a constant neural gain (ξ) was activated to induce neural couplings ($g_i(n)$ in Equation (17)) among the CPGs. The curves of $\gamma(n)$, ξ, $f_i(n)$, and $g_i(n)$ were of the RF leg. A video of this experiment can be viewed at `http://www.manoonpong.com/AdaptiveCommunications/videoS1.mp4`

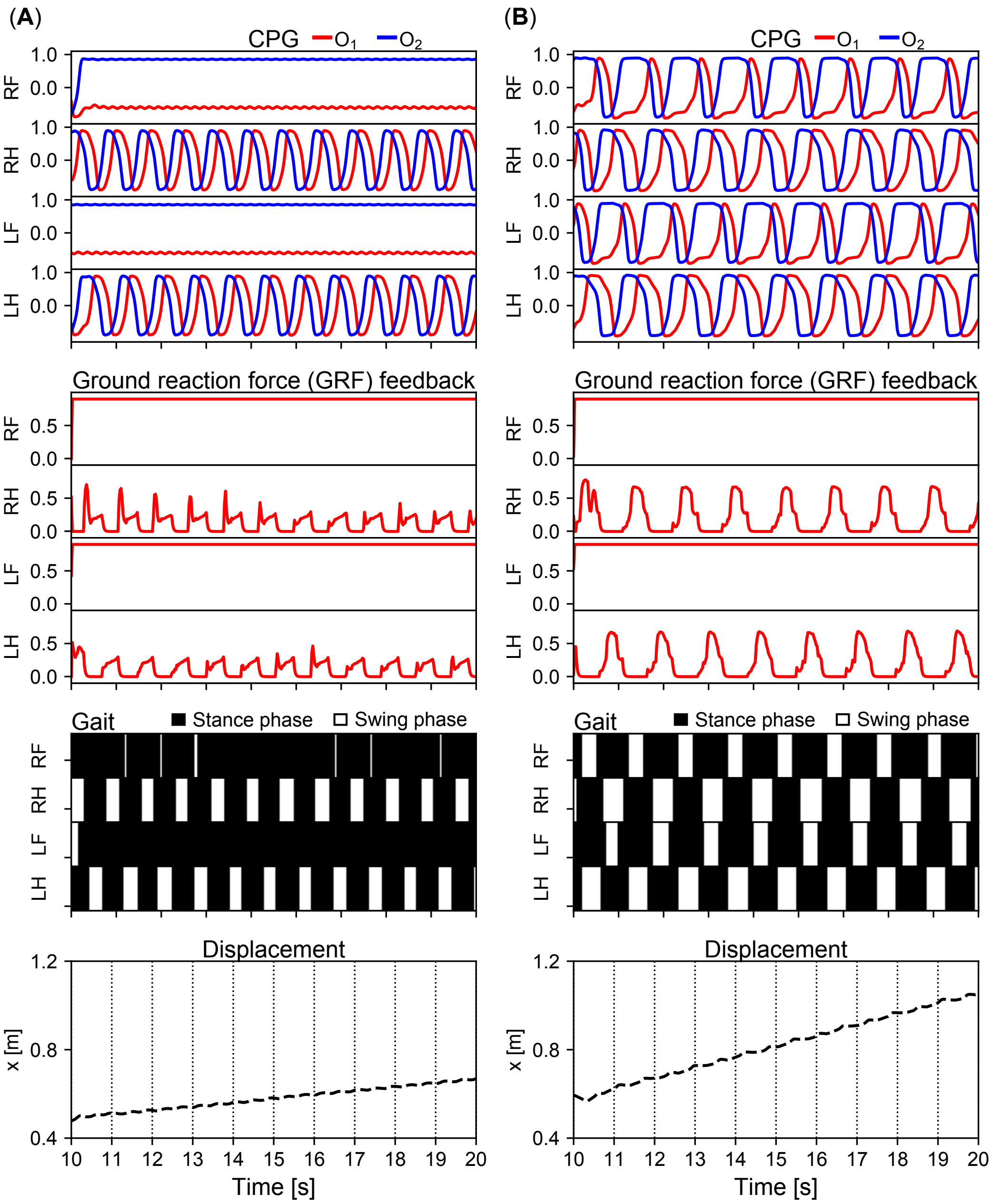

Figure S8: Real-time data of the robust locomotion (in S2). In this state, the sensory feedback (i.e., GRFs) of the front legs (i.e., RF and LF legs) was set to be a high constant value of 0.9 to simulate sensor malfunction. S2 was implemented under the adaptive neural control without and with ANC. The experiment results are shown in (A) and (B), respectively. In (A), the CPGs of the front legs stopped oscillating because sensor malfunction inhibited the CPGs' activities. Accordingly, the gait diagram in (A) shows that the front legs remained in the stance phase continuously, and that the displacement of the robot could not increase significantly, as the last plot shown in (A). In contrast, (B) demonstrates that all the CPGs continued to work, even if modulated by abnormal GRF feedback; the robot continued to move with a stable trot gait.

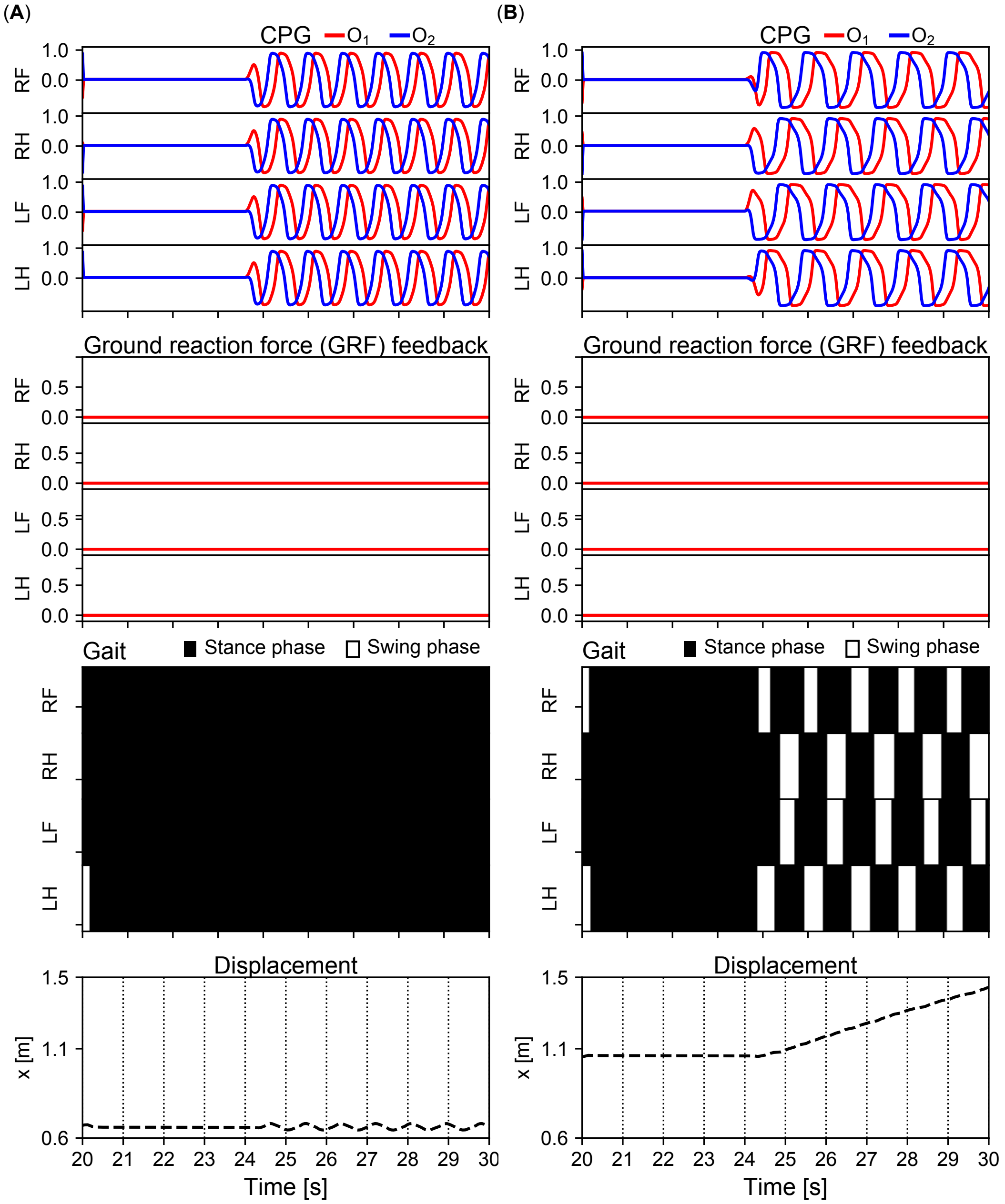

Figure S9: Real-time data of the memorized locomotion (in S3). In this state, the sensory feedback was removed to simulate sensory absence, and *MI* was first reset to zero to initialize the CPGs from the 20th s and then was set to 0.15 after the 24th s. The experiment was conducted using the control without and with ANC. The results are shown in (A) and (B), respectively. During the initialization period (20-24 s), the results are the same in (A) and (B). The CPG outputs were zero. All the GRF feedback to the CPGs was equal to zero and the robot was motionless. However, after the CPGs were reactivated (after the 24th s), (B) demonstrates that the robot started to move immediately. This is because the CPGs obtained the proper phase relationships from the neural communication. (A) shows that the robot continued to oscillate in place. This is because the CPGs had the same phase and the original relationships were not stored when neural communication were absent. Note that the difference in displacement values at the 20th s in (A) and (B) because the robot was started at different locations. In fact, the start points of (A) and (B) at the 20th s were the end displacement points of the experiment shown in Figure S8 (A) and (B), respectively.

5 COMPLEX ROBOT CONDITION DEFINITION

Table S5. The description of the four different robot conditions for self-organized locomotion experiments

Conditions	Description
C1 (normal condition)	This is a normal condition. It served as a baseline for comparison with other unexpected conditions (C2, C3, and C4).
C2 (noisy feedback)	The GRFs of four legs were added with Gaussian noise with a high amplitude of, e.g., 20% of the maximum value of the GRFs.
C3 (leg damage)	Arbitrary hip and knee joints (e.g., the joints of the right front leg) were fixed and the leg was unable to move during the experiment.
C4 (carrying a load)	The experiment robot (Lilibot) carried a payload (e.g., 0.6 kg load or 24% of the robot weight), and the load was arbitrarily placed, e.g., near the hind legs.

6 METRIC DEFINITIONS

6.1 Balance

Large-amplitude changes in body orientation (i.e., roll and pitch) are known to negatively affect the conservation of momentum and locomotion balance, thus increasing the risk of the robot falling (Iosa et al., 2012; Ferreira and Santos, 2017). Therefore, an unbiased estimator of standard deviations of the robot body roll and pitch angles were used to represent its balance during locomotion. The detailed definition is as follows:

$$\bar{q}_{r,p} = \frac{1}{N}\sum_{n=1}^{N} q(n)_{r,p}, \tag{S1}$$

$$q_{r,p}^{std} = \sqrt{\frac{1}{N-1}\sum_{n=1}^{N}(q(n)_{r,p} - \bar{q}_{r,q})^2}, \tag{S2}$$

where N is the total sample size of the body roll and pitch angles $q(n)_{r,q}$. Equations. (S1) and (S2) produce the mean and standard deviation of the roll and pitch angles during locomotion, respectively.

The standard deviation of the roll and pitch angles indicates the amplitude of oscillations within a period. The inverse of norm of the standard deviation is defined as a balance metric, as shown in Equation (S3). Thus, the larger the $balance$ value, the more stable the locomotion.

$$balance = \frac{1}{\sqrt{(q_r^{std})^2 + (q_p^{std})^2}}. \tag{S3}$$

6.2 Coordination

In addition to the body movement state, the robot's foot motions also significantly determine locomotion performance. Ideally, all robot feet should perform similar alternating movements from swing to stance. Thus, all legs should have the same duty factors during stable trotting. The duty factors were measured using the coordination metric, which is defined as follows:

$$\mu_i(m) = \frac{T_{swing}^i(m)}{T_{swing}^i(m) + T_{stance}^i(m)}, \tag{S4}$$

$$\bar{\mu}(m) = \frac{1}{4}\sum_{i=1}^{4}(\mu_i(m)), \tag{S5}$$

$$\mu_{std}(m) = \begin{cases} \sqrt{\frac{\sum_{i=1}^{4}(\mu_i(m) - \bar{\mu}(m))^2}{4}} & \forall m \leq M, i \leq 4, \exists \mu_i(m) \\ 0 & \text{otherwise} \end{cases}, \tag{S6}$$

$$coordination = \begin{cases} \frac{1}{\max\limits_{\{m \in M\}} (\mu_{std}(m))} & \exists \mu_{std}(m) \neq 0 \\ 0 & \text{otherwise} \end{cases}, \tag{S7}$$

where $T^i_{swing}(m)$ and $T^i_{stance}(m)$ in Equation (S4)) denote the swing and stance periods of the i-th leg during the m-th step, respectively. Thus, $\mu_i(m)$ defines the duty factor of the m-th step for the i-th leg, and the mean ($\bar{\mu(m)}$) and an unbiased estimator of standard deviation ($\mu_{std}(m)$) of the duty factor for the four legs (at the m-th step) are output to measure the irregularity/incongruity of the four leg movements. The inverse of the maximum standard deviation over several steps (i.e., M) was used to characterize the coordinated movements of the four legs. Therefore, the larger the $coordination$ value, the greater the coordination of a regular trot gait.

6.3 Cost of transport (COT)

The last metric is the *COT*. It is used to measure the energy efficiency of the formed self-organized locomotion over a period. The COT is described as follows:

$$\begin{cases} COT = \frac{E}{mgd}, \\ E = \sum_{j=1}^{12} \sum_{n=1}^{M} \frac{I_j(n)V_j(n)}{H}, \end{cases} \tag{S8}$$

where E is the energy consumption when the robot weighing mg travels over a distance d. The energy is calculated using the robot joint motor current $I_j(n)$ and voltage $V_j(n)$. M indicates the number of steps over the period. H is the update frequency of the experimental system.

7 STABILITY OF THE CONTROL SYSTEM UNDER DIFFERENT UPDATE FREQUENCIES

The stability of the control system under different update frequencies was also studied through decoupled CPGs phase convergence progression. The decoupled CPGs are a dynamic system whose convergence features impact the overall stability of the control system. Different update frequencies can cause different time-delay effects in the convergence progression of the decoupled CPGs, thereby influencing the control system's stability.

Figure S10 shows the phase portrait of the decoupled CPGs under different update frequencies. In the figure, each green line represents the average phase portrait of five repeated trials, where a trial lasted 35 s from the robot initial state (i.e., after placing the robot on the ground). In all the frequencies, the state variables of the dynamic system (green line) entirely converged to the desired state ((π, π, 0), blue point) from the initial state ((0, 0, 0), red point). More green lines are located near the desired state when the update frequency is high. This indicates that the system quickly converged to the desired point and stayed there. Besides, the repeated trials with the same update frequency may show different convergence progression because of the sensitivity of the system to its motion dynamics which was highly variable. Although the dynamic system's convergence progression exhibited different projections, it always converged to its stable state (π,π,0) where a stable gait was formed.

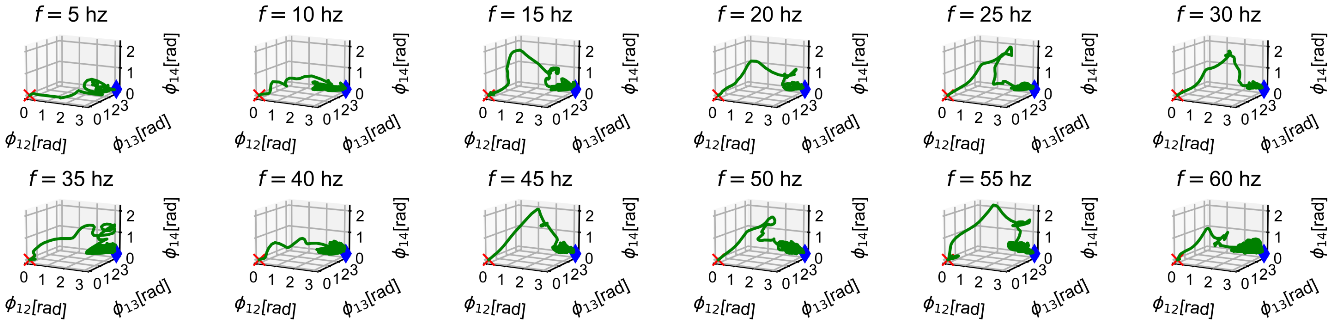

Figure S10: Average phase portrait of the decoupled CPGs modulated by the APNC under different update frequencies of the control system. At all frequencies, the state variables of the dynamic system (green points) entirely converged to the desired state ((π, π, 0), blue point) from the initial state ((0, 0, 0), red point). More green points are located near the desired state when the update frequency is high, indicating that the system quickly converges to the desired point. For each update frequency, we used five trials to calculate the average phase portrait. In this investigation, the *MI* value defining the robot walking frequency was set to 0.08.

8 COMPARISON BETWEEN PROPOSED APNC AND STATE-OF-ART METHODS

A comparison between the proposed adaptive neural control and state-of-art methods (Owaki et al., 2017; Aoi et al., 2012; Miguel-Blanco and Manoonpong, 2020; Fukuhara et al., 2018; Barikhan et al., 2014; Buchli and Ijspeert, 2008) for fast self-organized locomotion generation (i.e., obtaining a gait in less than a minute and without robot kinematics and environmental models, as well as predefined interlimb coordination) was conducted. As can be observed from Table S6, the proposed method was successful in this regard not only on even terrains (similar to the others) but also on uneven terrains owing to the APC. It also achieved motor memory through the ANC for gait recovery and robust locomotion to deal with sensory feedback malfunction. Furthermore, it enabled spontaneous change in the walking speed and direction, which is robot maneuverability. Last but not least, our approach is also general and flexible. Our preliminary result shows that it can be applied to a large-sized quadruped robot (Laikago) for self-organized locomotion. A video of this preliminary experiment can be viewed at `https://www.manoonpong.com/AdaptiveCommunications/videoS2.mp4`.

Table S6. Comparison between our proposed adaptive neural control and state-of-art methods (Owaki et al., 2017; Aoi et al., 2012; Miguel-Blanco and Manoonpong, 2020; Fukuhara et al., 2018; Barikhan et al., 2014; Buchli and Ijspeert, 2008) for fast self-organized locomotion generation

Method	Self-organized locomotion	Motor memory	Flat terrain	Rough terrain	Maneuverability
Our adaptive neural control with the APC and ANC	yes	yes	yes	yes	yes
Miguel-Blanco et al., 2020 (Miguel-Blanco and Manoonpong, 2020)	yes	no	yes	no	no
Fukuhara et al., 2018 (Fukuhara et al., 2018)	yes	no	yes	no	no
Shaker et al., 2014 (Barikhan et al., 2014)	yes	no	yes	no	no
Owaki et al., 2017 (Owaki et al., 2017)	yes	no	yes	no	no
Aoi et al., 2012 (Aoi et al., 2012)	yes	no	yes	no	no
Jonas et al., 2008 (Buchli and Ijspeert, 2008)	yes	no	yes	no	no

9 ABBREVIATIONS AND ACRONYMS

ACI = Adaptive control input.
APC = Adaptive physical communication.
APNC = Adaptive physical and neural communication.
ANC = Adaptive neural communication.
ANN = Artificial neural network.
APR = Acquisition of phase relationships
CPG(s) = Central pattern generator(s).
DL = Dual-rate learner.
EPR = Estimation of phase relationships.
EA = Evolutionary algorithm.
FM = Forward model.
GRFs = Ground reaction force(s).

IMU = Inertial measurement unit.
LF = Left front leg.
LH = Left hind leg.
ML = Machine learning.
MN(s) = Motor neuron(s).
PR = Phase resetting.
RF = Right front leg.
RH = Right hind leg.
ROS = Robot operation system.
RL = Reinforcement learning.
SFM = sensory feedback mechanism.
S1 = State 1.
S2 = State 2.
S3 = State 3.

REFERENCES

Aoi, S., Amano, T., Fujiki, S., Senda, K., and Tsuchiya, K. (2021). Fast and slow adaptations of interlimb coordination via reflex and learning during split-belt treadmill walking of a quadruped robot. *Frontiers in Robotics and AI* 8. doi:10.3389/frobt.2021.697612

Aoi, S., Egi, Y., Sugimoto, R., Yamashita, T., Fujiki, S., and Tsuchiya, K. (2012). Functional roles of phase resetting in the gait transition of a biped robot from quadrupedal to bipedal locomotion. *IEEE Transactions Robotics* 28, 1244–1259. doi:10.1109/TRO.2012.2205489

Aoi, S., Fujiki, S., Yamashita, T., Kohda, T., Senda, K., and Tsuchiya, K. (2011). Generation of adaptive splitbelt treadmill walking by a biped robot using nonlinear oscillators with phase resetting. In *2011 IEEE/RSJ International Conference on Intelligent Robots and Systems* (IEEE), 2274–2279

Aoi, S., Yamashita, T., Ichikawa, A., and Tsuchiya, K. (2010). Hysteresis in gait transition induced by changing waist joint stiffness of a quadruped robot driven by nonlinear oscillators with phase resetting. In *2010 IEEE/RSJ International Conference on Intelligent Robots and Systems* (Taipei, Taiwan: IEEE), 1915–1920

Barikhan, S. S., Wörgötter, F., and Manoonpong, P. (2014). Multiple decoupled cpgs with local sensory feedback for adaptive locomotion behaviors of bio-inspired walking robots. In *From Animals to Animats 13* (Cham: Springer International Publishing), 65–75

Buchli, J. and Ijspeert, A. J. (2008). Self-organized adaptive legged locomotion in a compliant quadruped robot. *Autonomous Robots* 25, 331–347

Ferreira, C. and Santos, C. P. (2017). A sensory-driven controller for quadruped locomotion. *Biological Cybernetics* 111, 49–67

Fukuhara, A., Owaki, D., Kano, T., Kobayashi, R., and Ishiguro, A. (2018). Spontaneous gait transition to high-speed galloping by reconciliation between body support and propulsion. *Advanced Robotics* 32, 794–808. doi:10.1080/01691864.2018.1501277

Iosa, M., Marro, T., Paolucci, S., and Morelli, D. (2012). Stability and harmony of gait in children with cerebral palsy. *Research in Developmental Disabilities* 33, 129–135

Kano, T., Yoshizawa, R., and Ishiguro, A. (2017). Tegotae-based decentralised control scheme for autonomous gait transition of snake-like robots. *Bioinspiration & biomimetics* 12, 046009

Miguel-Blanco, A. and Manoonpong, P. (2020). General distributed neural control and sensory adaptation for self-organized locomotion and fast adaptation to damage of walking robots. *Frontiers in Neural Circuits* 14, 46. doi:10.3389/fncir.2020.00046

Nomura, T., Kawa, K., Suzuki, Y., Nakanishi, M., and Yamasaki, T. (2009). Dynamic stability and phase resetting during biped gait. *Chaos: An Interdisciplinary Journal of Nonlinear Science* 19, 026103

Owaki, D., Goda, M., Miyazawa, S., and Ishiguro, A. (2017). A minimal model describing hexapedal interlimb coordination: The tegotae-based approach. *Frontiers in Neurorobotics* 11, 29. doi:10.3389/fnbot.2017.00029

Owaki, D., Horikiri, S.-y., Nishii, J., and Ishiguro, A. (2021). Tegotae-based control produces adaptive inter-and intra-limb coordination in bipedal walking. *Frontiers in neurorobotics* 15, 47

Rohmer, E., Singh, S. P. N., and Freese, M. (2013). V-rep: A versatile and scalable robot simulation framework. In *2013 IEEE/RSJ International Conference on Intelligent Robots and Systems*. 1321–1326. doi:10.1109/IROS.2013.6696520

Smith, M. A., Ghazizadeh, A., and Shadmehr, R. (2006). Interacting adaptive processes with different timescales underlie short-term motor learning. *PLoS Biology* 4, e179

Sun, T., Xiong, X., Dai, Z., and Manoonpong, P. (2020). Small-sized reconfigurable quadruped robot with multiple sensory feedback for studying adaptive and versatile behaviors. *Frontiers in Neurorobotics* 14, 14

Sun, T., Xiong, X., Dai, Z., Owaki, D., and Manoonpong, P. (2021). A comparative study of adaptive interlimb coordination mechanisms for self-organized robot locomotion. *Frontiers in Robotics and AI* 8, 86. doi:10.3389/frobt.2021.638684
